# Defects in meiotic recombination delay progression through pachytene in *Tex19.1*^*−/−*^ mouse spermatocytes

**DOI:** 10.1007/s00412-018-0674-9

**Published:** 2018-06-16

**Authors:** James H. Crichton, David Read, Ian R. Adams

**Affiliations:** MRC Human Genetics Unit, MRC Institute of Genetics and Molecular Medicine, University of Edinburgh, Western General Hospital, Crewe Road, Edinburgh, EH4 2XU UK

**Keywords:** Meiosis, Recombination, Pachytene, Tex19.1, Spo11, Checkpoint

## Abstract

Recombination, synapsis, chromosome segregation and gene expression are co-ordinately regulated during meiosis to ensure successful execution of this specialised cell division. Studies with multiple mutant mouse lines have shown that mouse spermatocytes possess quality control checkpoints that eliminate cells with persistent defects in chromosome synapsis. In addition, studies on *Trip13*^*mod/mod*^ mice suggest that pachytene spermatocytes that successfully complete chromosome synapsis can undergo meiotic arrest in response to defects in recombination. Here, we present additional support for a meiotic recombination-dependent checkpoint using a different mutant mouse line, *Tex19.1*^*−/−*^. The appearance of early recombination foci is delayed in *Tex19.1*^*−/−*^ spermatocytes during leptotene/zygotene, but some *Tex19.1*^*−/−*^ spermatocytes still successfully synapse their chromosomes and we show that these spermatocytes are enriched for early recombination foci. Furthermore, we show that patterns of axis elongation, chromatin modifications and histone H1t expression are also all co-ordinately skewed towards earlier substages of pachytene in these autosomally synapsed *Tex19.1*^*−/−*^ spermatocytes. We also show that this skew towards earlier pachytene substages occurs in the absence of elevated spermatocyte death in the population, that spermatocytes with features of early pachytene are present in late stage *Tex19.1*^*−/−*^ testis tubules and that the delay in histone H1t expression in response to loss of *Tex19.1* does not occur in a *Spo11* mutant background. Taken together, these data suggest that a recombination-dependent checkpoint may be able to modulate pachytene progression in mouse spermatocytes to accommodate some types of recombination defect.

**Electronic supplementary material:**

The online version of this article (10.1007/s00412-018-0674-9) contains supplementary material, which is available to authorized users.

## Introduction

Meiosis is a central feature of the life cycle of sexually reproducing organisms that requires co-ordinated regulation of multiple distinct processes including recombination, chromosome synapsis, chromosome segregation and changes in gene expression, to generate haploid germ cells (Baudat et al. 2013; MacLennan et al. 2015; Cahoon and Hawley 2016). At least some of these processes are monitored by checkpoints, which can co-ordinate meiotic events and can also provide a quality control mechanism to eliminate cells that do not execute some aspects of meiosis correctly (Subramanian and Hochwagen 2014). Checkpoints also operate in somatic cells to improve the fidelity of the mitotic cell cycle, though some cell types appear to lack some checkpoints, possibly in contexts where checkpoint-induced delays might cause asynchrony and developmental problems (Hartwell and Weinert 1989). In meiotic spermatocytes in the mouse testis, there is strong evidence that extensive defects in meiotic chromosome synapsis can activate a checkpoint that triggers cell death during pachytene (Mahadevaiah et al. 2008). This synapsis checkpoint is caused by asynapsed chromosomes sequestering the transcriptional silencing machinery away from the heterologous sex chromosomes, causing defective meiotic sex chromosome inactivation (MSCI) and inappropriate expression of sex chromosome-encoded gene products (Burgoyne et al. 2009). In contrast, mouse oocytes, which do not normally exhibit MSCI, have a more heterogeneous response to chromosome asynapsis which potentially reflects transcriptional silencing of essential meiotic genes on the asynapsed chromosomes themselves (Burgoyne et al. 2009; Cloutier et al. 2015). These synapsis checkpoints can induce cell death to remove abnormal meiotic cells from the population and prevent them progressing further in gametogenesis.

In addition to synapsis checkpoints, mouse oocytes and spermatocytes can also undergo meiotic arrest in response to recombination defects (Bolcun-Filas et al. 2014; Pacheco et al. 2015; Marcet-Ortega et al. 2017). However, the tight coupling between recombination and synapsis means that many meiotic recombination mutants also have severe synapsis defects that activate the synapsis checkpoint (Mahadevaiah et al. 2008), making it difficult to study the effects of perturbed recombination on meiotic progression in the absence of confounding effects of asynapsis. Despite this, analysis of different asynapsed recombination-defective mouse spermatocytes has revealed that the presence of DSBs and/or recombination intermediates can influence pachytene gene expression. *Spo11*^*−/−*^ spermatocytes, which fail to form meiotic DSBs and have synapsis defects, express the mid/late pachytene marker histone H1t before arresting (Barchi et al. 2005). In contrast, *Dmc1*^*−/−*^ spermatocytes, which have defects in the early stages of recombination and exhibit persistent unrepaired DSBs and synapsis defects, lack or have greatly reduced histone H1t expression (Barchi et al. 2005; Mahadevaiah et al. 2008). However, *Msh5*^*−/−*^ spermatocytes, which are defective in later stages of meiotic recombination but still have synapsis defects, successfully express histone H1t despite the persistence of unrepaired DSBs (Barchi et al. 2005; Mahadevaiah et al. 2008). Therefore, although each of these mutants has severe chromosome synapsis defects, the specific recombination defects and/or the presence of specific recombination intermediates appears to influence progression through pachytene as assessed by expression of histone H1t.

Further insights into the relationship between recombination and pachytene progression in spermatocytes have come from mice carrying moderate severity mutations in *Trip13* (Pacheco et al. 2015; Marcet-Ortega et al. 2017). *Trip13* encodes an AAA+ ATPase implicated in regulating HORMAD proteins (Wojtasz et al. 2009), which in turn have diverse roles in DSB processing, synaptonemal complex formation and monitoring chromosome synapsis (Wojtasz et al. 2009, 2012; Shin et al. 2010; Daniel et al. 2011). Severe mutations in *Trip13* result in asynapsis, but moderate severity hypomorphic *Trip13* mutant (*Trip13*^*mod/mod*^) spermatocytes successfully synapse their chromosomes and exhibit defects in maturation of recombination foci (Li et al. 2007; Roig et al. 2010). These *Trip13*^*mod/mod*^ spermatocytes have been used to study the consequences of recombination defects independently of defects in synapsis (Pacheco et al. 2015; Marcet-Ortega et al. 2017). Fully synapsed pachytene *Trip13*^*mod/mod*^ spermatocytes activate DNA damage signalling pathways and undergo pachytene arrest and cell death, largely in pachytene cells that do not express the mid/late pachytene marker histone H1t (Li et al. 2007; Roig et al. 2010; Pacheco et al. 2015; Marcet-Ortega et al. 2017). Combining the *Trip13*^*mod/mod*^ mutation with mutations in the *Chk2*-dependent DNA damage signalling pathway allows spermatocytes to progress to a histone H1t-positive mid/late pachytene stage, where they exhibit MSCI failure and undergo cell death (Pacheco et al. 2015; Marcet-Ortega et al. 2017). Similar to *Trip13*^*mod/mod*^ mice, mice with a conditional germline deletion of *Hus1*, a component of the 9-1-1 complex known to respond to DNA damage in somatic cells, also possess spermatocytes that fully synapse their autosomes and have elevated levels of unrepaired DNA damage in pachytene (Lyndaker et al. 2013). In contrast to *Trip13*^*mod/mod*^ spermatocytes, *Hus1* conditional knockout spermatocytes advance beyond pachytene into diplotene despite the presence of unrepaired DNA damage (Lyndaker et al. 2013). This could reflect differences in the recombination defects in these two mutants or could be related to reduced *Chk2*-dependent DNA damage signalling in the *Hus1* conditional knockout spermatocytes (Lyndaker et al. 2013). However, it is not clear if recombination-dependent checkpoints in mammalian spermatocytes only act as quality control mechanisms to eliminate defective spermatocytes, or whether these checkpoints can alter meiotic progression to accommodate repair of less severe recombination defects.

*Tex19.1* was originally isolated as a testis-expressed gene (Wang et al. 2001) and is a member of a mammal-specific gene family of *TEX19* genes that has duplicated in rodents to generate *Tex19.1* and *Tex19.2* (Kuntz et al. 2008). *Tex19.1* expression is regulated primarily and causally by promoter DNA methylation (Hackett et al. 2012), and *Tex19.1* RNA is the target for the RNA-binding protein and infertility factor DAZL (Reynolds et al. 2005; Chen et al. 2011). *Tex19.1* has been proposed to encode a nuclear protein with a role in regulating pluripotency or stem cell self-renewal (Kuntz et al. 2008). However, studies using antibodies specific for endogenous TEX19.1 protein and that functionally characterise the in vivo role of endogenous *Tex19.1* show that TEX19.1 is predominantly present in the cytoplasm in the mouse germline and that it has roles in meiosis and spermatogenesis, placenta development and suppression of specific retrotransposon-encoded transcripts (Öllinger et al. 2008; Yang et al. 2010; Reichmann et al. 2012, 2013; Tarabay et al. 2013). TEX19.1 protein interacts with the E3 ubiquitin ligase UBR2 (Yang et al. 2010), requires *Ubr2* for its stability (Yang et al. 2010), directly interacts with retrotransposon-encoded proteins to promote their ubiquitin-dependent proteolysis (MacLennan et al. 2017) and prevents retrotransposons from mobilising to new locations in the genome (MacLennan et al. 2017). *Tex19.1* and *Ubr2* also both have a role in progression through meiosis (Kwon et al. 2003; Öllinger et al. 2008), and *Tex19.1*^*−/−*^ and *Ubr2*^*−/−*^ spermatocytes exhibit similar defects in early meiotic recombination (Crichton et al. 2017). It is not clear whether the functions of *Tex19.1* and *Ubr2* in meiotic progression are related to their roles in retrotransposon regulation or reflect a more direct role in regulating meiotic recombination (Crichton et al. 2017). Around two thirds of *Tex19.1*^*−/−*^ pachytene spermatocytes exhibit defects in chromosome synapsis, which appears to induce cell death at this stage of meiosis (Öllinger et al. 2008; Yang et al. 2010; Crichton et al. 2017). These synapsis defects are associated with earlier defects in meiotic recombination (Crichton et al. 2017). *Tex19.1*^*−/−*^ spermatocytes have reduced numbers of early recombination foci during leptotene, and similar to hypomorphic *Spo11* spermatocytes (Kauppi et al. 2013), additional early recombination foci are generated during zygotene (Crichton et al. 2017). Despite this delay in accumulation of early recombination foci, approximately one third of *Tex19.1*^*−/−*^ pachytene spermatocytes synapse their autosomes completely (Öllinger et al. 2008; Crichton et al. 2017). Some *Tex19.1*^*−/−*^ spermatocytes progress through meiosis to metaphase I, and the majority of the chromosomes in metaphase I *Tex19.1*^*−/*−^ spermatocytes are present in chiasmata-containing bivalents suggesting that at least some of the recombination that occurs in *Tex19.1*^*−/*−^ spermatocytes successfully generates crossovers (Öllinger et al. 2008). However, there are also some univalent chromosomes in metaphase I *Tex19.1*^*−/−*^ spermatocytes that may trigger additional cell death at this stage (Öllinger et al. 2008). Therefore, despite the delay in early recombination, some *Tex19.1*^*−/−*^ spermatocytes successfully complete chromosome synapsis and progress through the first meiotic prophase. These autosomally synapsed *Tex19.1*^*−/−*^ spermatocytes therefore provide a system to complement studies on *Trip13*^*mod/mod*^ spermatocytes and investigate how delays in recombination might influence progression through pachytene in the absence of the confounding effects of asynapsis.

Here, we show that progression through pachytene is perturbed in autosomally synapsed *Tex19.1*^*−/−*^ spermatocytes. We show that recombination dynamics, chromosome axis elongation, chromatin modifications and histone H1t expression are co-ordinately altered in *Tex19.1*^*−/−*^ spermatocytes and that the altered pachytene progression of *Tex19.1*^*−/−*^ spermatocytes depends on *Spo11*. These findings are consistent with the hypothesis that meiotic recombination is monitored by a checkpoint-type mechanism in mouse spermatocytes that can delay multiple aspects of pachytene progression in order to accommodate cells with altered recombination kinetics.

## Materials and methods

### Mice

*Tex19.1*^*−/−*^ animals on a C57BL/6 genetic background were bred and genotyped as described (Öllinger et al. 2008). *Spo11*^*+/−*^ heterozygous mice (Baudat et al. 2000) on a C57BL/6 genetic background (Mahadevaiah et al. 2008) were inter-crossed with *Tex19.1*^*+/−*^ mice. Adult male mice between 6 weeks and 6 months old were used for the analyses in this paper unless otherwise stated. For post-natal staging, the day of birth was designated 1 day post-partum (1 dpp). Animals were culled by cervical dislocation. *Tex19.1*^*+/+*^ and *Tex19.1*^*+/−*^ animals have no difference in spermatogenesis as assessed by epididymal sperm counts (Öllinger et al. 2008) and no difference in abundance of recombination foci during early prophase (Crichton et al. 2017); therefore, data from these control genotypes were pooled as *Tex19.1*^*+/±*^. *Tex19.1*^*+/±*^ littermates were used as controls for *Tex19.1*^*−/−*^ mice.

### Immunostaining meiotic chromosome spreads

Chromosome spreads were prepared as described by Peters et al. (1997) or by Costa et al. (2005) and stored at − 80 °C until use. After thawing, chromosome spreads were blocked with PBS containing 0.15% BSA, 0.1% Tween-20 and 5% goat serum. Primary antibodies were also diluted in PBS containing 0.15% BSA, 0.1% Tween-20 and 5% goat serum as indicated in Online Resource 1. Alexa Fluor-conjugated secondary antibodies (Invitrogen) were used at a 1:500 dilution, and DNA was stained by including 2 ng/μl 4,6-diamidino-2-phenylidole (DAPI) in the secondary antibody incubations. Slides were mounted in 90% glycerol, 10% PBS and 0.1% *p*-phenylenediamine, and images captured with iVision, IPLab software (BioVision Technologies) or Micro-Manager (Open Imaging) software using an Axioplan II fluorescence microscope (Carl Zeiss) equipped with motorised colour filters. At least three experimental and three control animals were analysed for each experiment. RAD51 and RPA recombination foci were imaged by capturing z-stacks using a piezoelectrically driven objective mount (Physik Instrumente) controlled with Volocity software (PerkinElmer). These images were deconvolved using Volocity, and a 2D image was generated in Fiji (Schindelin et al. 2012).

Nuclei were staged by immunostaining for the axial/lateral element marker SYCP3 (Lammers et al. 1994). Leptotene nuclei were recognised by incomplete, fragmented axial elements and a lack of synapsis indicated by the absence of co-localisation between SYCP3 and the transverse filament marker SYCP1 (Meuwissen et al. 1992). Zygotene nuclei were identified by extensive axial element formation and partial synapsis. Pachytene nuclei were identified by complete co-localisation of SYCP3 and either SYCP1 or the central element marker SYCE2 (Costa et al. 2005) on all 19 autosomes, or by the presence of 19 bold SYCP3 axes in addition to two paired or unpaired sex chromosome axes of unequal length. The synapsis status of the X and Y chromosomes was not considered in these analyses as the timing and kinetics of sex chromosome synapsis differs from autosomes (Kauppi et al. 2011). Asynapsed pachytene *Tex19.1*^*−/−*^ nuclei (Öllinger et al. 2008) were distinguished from zygotene nuclei by the presence of at least one completely synapsed pair of autosomes and at least one pair of autosomes exhibiting asynapsis along at least half its length (Crichton et al. 2017). Diplotene nuclei were recognised by incomplete synapsis, bold axial element staining and enrichment of SYCP3 at axis termini. Zygotene-like nuclei in *Spo11*^*−/−*^ mice were identified by the presence of fully formed axial elements.

DMC1, RAD51, RPA and MLH1 foci were counted as recombination foci when they overlapped a chromosome axis, and the foci counts reported refer to autosomal foci only. Nuclei were classed as MLH1-positive if they contained more than ten axial MLH1 foci. For γH2AX foci, prominent foci associated with SYCP3-stained axes were scored. For RBMY and histone H1t scoring, nuclei were imaged with fixed exposures, and the image filenames were then randomised by a computer script and scored blind as positive or negative. For analysis of FK2 staining, axial autosome staining is significantly less intense than the signal in the XY body, and image contrast was adjusted appropriately to score each of these parameters. Axis length was measured using NeuronJ (Meijering et al. 2004) as described previously (Schoenmakers et al. 2008). All scoring of immunostained chromosome spreads was performed blind on images after randomisation of filenames by computer script. Data were analysed in R (R Core Team 2016), and means are reported ± standard deviation. Nuclei were typically obtained from at least three animals for each genotype; for categorical data, stacked bar graphs represent proportions of all nuclei scored, while the means and interquartile ranges indicate the variation between animals of the same genotype.

### Combined immunofluorescence and fluorescence in situ hybridisation (immunoFISH) on chromosome spreads

Slides were washed briefly in PBS, 2 × SSC and then denatured in 70% formamide in 2 × SSC for 30 min at 80 °C. Ten microliters of fluorescently labelled Mouse Whole Chromosome Painting Probes for chromosome 18 or chromosome 19 (Carl Zeiss Ltd.) were denatured at 70 °C for 5 min, re-annealed for 15 min at 37 °C and hybridised under a coverslip overnight at 37 °C. Slides were then washed four times in 2 × SSC for 3 min at 45 °C each, four times in 0.1 × SSC for 3 min at 60 °C each, and then with 2 × SSC/0.1% Tween-20 for 3 min at room temperature. Axial elements were then stained with 1:500 mouse anti-SYCP3 (Abcam, Online Resource 1) as described for immunostaining meiotic chromosome spreads. Chromosome domains were judged either to be separate if there were two domains visible that shared no overlap, or overlapping if there was a single continuous domain.

### Immunostaining testis sections

Testes were fixed in Bouin’s solution (Sigma) for 3 h and embedded in wax before cutting 5 μm sections. Slides were de-waxed in xylene, then rehydrated through an alcohol series. Antigen retrieval was achieved by incubating slides in Target Retrieval Solution (Dako) and autoclaving at 121 °C for 20 min, then allowing to cool. Immunohistochemistry was performed using 1:1000 guinea pig anti-histone H1t primary antibodies (Online Resource 1), followed by 1:2000 rabbit anti-guinea pig-HRP (Invitrogen) bridging antibody then visualised with an EnVision+ System-HRP (DAB) kit for use with rabbit primary antibodies (Dako) according to the manufacturer’s instructions. Slides were counterstained in Harris haematoxylin. Seminiferous tubules were staged as described (Ahmed and de Rooij 2009). Images were acquired using a Micropublisher 5MP cooled colour CCD camera (Qimaging, Surrey, BC, Canada) mounted on a Zeiss Axioplan II fluorescence microscope with Plan-neofluar or Plan Apochromat objectives (Carl Zeiss, Cambridge, UK). Image capture was performed using Micromanager (Open Imaging). Alternatively, immunofluorescence was performed on de-waxed and rehydrated testis sections after antigen retrieval. Immunofluorescence was as described for chromosome spreads using 1:2000 mouse anti-γH2AX and 1:500 rabbit anti-SYCP3 (LS-Bio) primary antibodies (Online Resource 1). Fluorescent staining on sections was imaged by capturing z-stacks using a piezoelectrically driven objective mount (Physik Instrumente) controlled with NIS Elements software (Nikon). These images were deconvolved using Volocity, and a 2D image was generated in Fiji (Schindelin et al. 2012). Tubules at stage VIII were identified by SYCP3 aggregates in pre-leptotene spermatocytes (Nakata et al. 2015) and distinguished from stage IX tubules by the absence of үH2AX staining in leptotene spermatocytes (Mahadevaiah et al. 2001; Ahmed and de Rooij 2009).

### TUNEL staining

TUNEL staining to assess cell death was performed on wax sections of testes fixed in 4% paraformaldehyde in PBS. Cells were stained using a DeadEnd Fluorometric TUNEL System (Promega) following the manufacturer’s guidelines. Cells were counterstained with DAPI to identify seminiferous tubules, and the number of TUNEL-positive nuclei within seminiferous tubules counted after image filenames was randomised by computer script.

## Results

### Early recombination markers are enriched in autosomally synapsed *Tex19.1*^*−/−*^ pachytene spermatocytes

We have previously shown that *Tex19.1*^*−/−*^ spermatocytes have defects in early meiotic recombination (Crichton et al. 2017). The number of DMC1-containing recombination foci in *Tex19.1*^*−/−*^ spermatocytes is reduced to around 30% of those present in littermate controls during leptotene, but increases to reach 87% of control levels during zygotene (Crichton et al. 2017). Thus, a significant number of recombination foci are being generated during zygotene rather than leptotene in *Tex19.1*^*−/−*^ spermatocytes, possibly due to stimulation of *Spo11*-dependent recombination on asynapsed chromatin (Kauppi et al. 2013). These early recombination defects are accompanied by autosomal synapsis defects in approximately two thirds of the *Tex19.1*^*−/−*^ pachytene spermatocytes (Crichton et al. 2017), but the remaining fully synapsed pachytene spermatocytes presumably possessed sufficient recombination foci to promote a successful homology search, and do not have any confounding autosomal asynapsis that would interfere with analysing the effects of delayed recombination on meiotic progression. We have therefore used these autosomally synapsed pachytene *Tex19.1*^*−/−*^ nuclei to investigate whether delayed recombination might subsequently affect progression through the pachytene stage of meiosis.

**Fig. 1:**
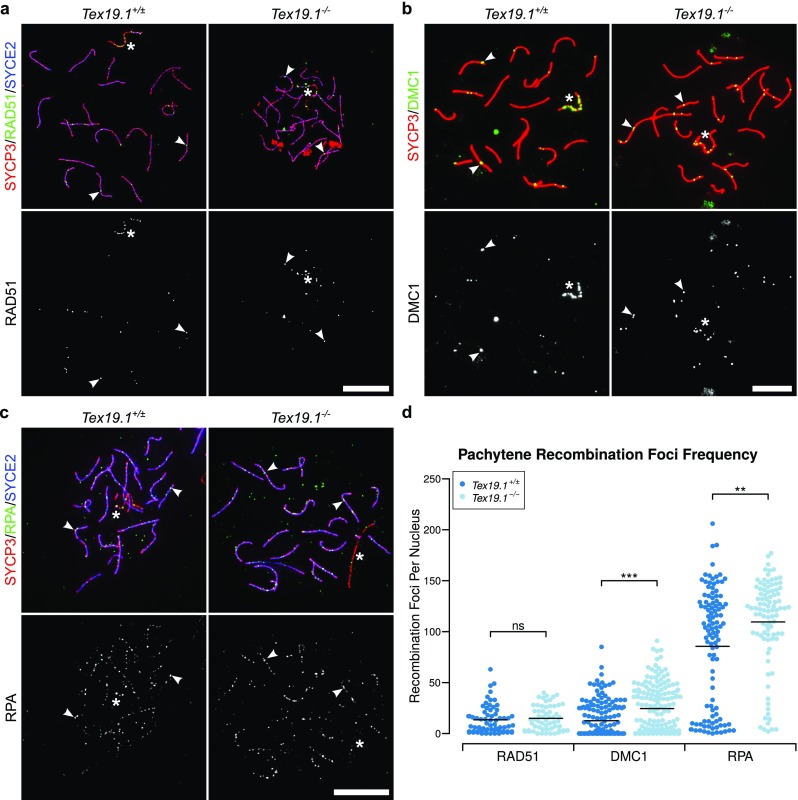
Autosomally synapsed pachytene *Tex19.1*^*−/−*^ spermatocytes have increased numbers of early meiotic recombination foci. **a**–**c** Immunostaining for RAD51 (**a**), DMC1 (**b**) and RPA (**c**) recombination proteins in *Tex19.1*^*+/±*^ and *Tex19.1*^*−/−*^ autosomally synapsed pachytene spermatocyte chromosome spreads. The recombination proteins are shown in green; SYCP3 (**a**–**c** red) and SYCE2 (**a**, **c** blue) mark the lateral and central elements of the synaptonemal complex, respectively. Single channel greyscale images for the recombination foci are also shown; foci co-localising with axes were scored as recombination foci. Asterisks indicate sex chromosomes, and arrowheads indicate example recombination foci. Scale bars 10 μm. **d** Scatterplots showing the number of axial recombination foci in autosomally synapsed pachytene nuclei. Means are indicated by horizontal lines. Mean foci frequencies are 13 ± 14, 15 ± 13, 13 ± 17, 24 ± 24, 86 ± 57 and 110 ± 44 from left to right across the plot. Numbers of nuclei analysed were 66, 60, 165, 163, 109 and 64 from a total of at least 3 experimental or 3 control animals for each recombination protein. Foci counts were compared between genotypes using a Mann-Whitney *U* test; asterisks denote significance (**p < 0.01, ***p < 0.001), ns indicates no significant difference (*p* > 0.05)

To investigate the kinetics of maturation of recombination foci in autosomally synapsed pachytene *Tex19.1*^*−/−*^ spermatocytes, we immunostained chromosome spreads from testes from adult *Tex19.1*^*−/−*^ mice, and from *Tex19.1*^*+/±*^ littermate controls, with antibodies to DMC1, RAD51 and RPA (Fig. 1a–c). DMC1 and RAD51 mark early recombination foci and dissociate as recombination foci mature during pachytene (Moens et al. 2002). RPA continues to mark recombination foci after DMC1 and RAD51 dissociate, but is also eventually lost during pachytene as recombination foci mature (Moens et al. 2002). Pachytene nuclei with complete autosomal synapsis were identified by immunostaining for synaptonemal complex components, and autosomally asynapsed pachytene nuclei, which are abundant in *Tex19.1*^*−/−*^ testes, were excluded from this analysis. Control pachytene *Tex19.1*^*+/±*^ spermatocytes had 13 ± 14 RAD51 foci, 13 ± 17 DMC1 foci and 86 ± 57 RPA foci on their autosomes (Fig. 1d), comparable to other studies (Adelman and Petrini 2008; Roig et al. 2010). Interestingly, mean autosomal DMC1 and RPA foci in autosomally synapsed *Tex19.1*^*−/−*^ pachytene spermatocytes were significantly increased by 95 and 28%, respectively (Fig. 1d). Violin plots of these data (Online Resource 1) suggest bimodal distributions for both DMC1 and RPA, with one population of pachytene nuclei containing relatively high numbers of DMC1 or RPA foci, and a second population containing few or no DMC1 or RPA foci. The increase in RPA foci number in autosomally synapsed *Tex19.1*^*−/−*^ pachytene spermatocytes appears to primarily reflect a shift towards the abundant foci population, whereas the increase in DMC1 foci number reflects both a shift towards the abundant foci population and an increase in the number of DMC1 foci in that population (Online Resource 1). RAD51 foci were not significantly increased in *Tex19.1*^*−/−*^ spermatocytes (Fig. 1d), although it is not clear if this reflects a genuine difference between RAD51 and its meiotic homologue DMC1 rather than technical differences in antibody sensitivity. Regardless, the increased frequency of DMC1 and RPA foci in pachytene *Tex19.1*^*−/−*^ spermatocytes indicates that, despite successful autosomal synapsis, these cells possess an abnormally high frequency of recombination foci participating in early stages of meiotic recombination.

**Fig. 2:**
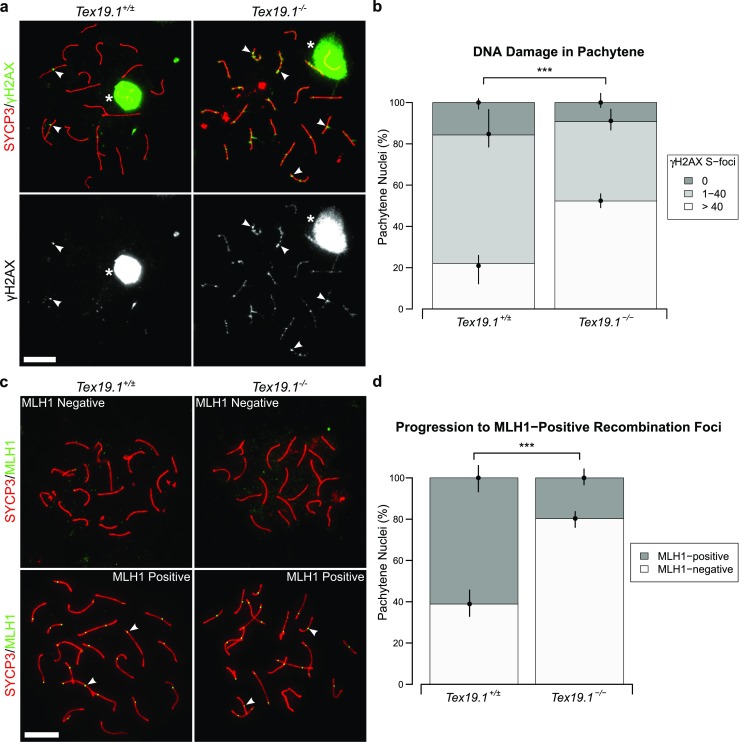
Autosomally synapsed pachytene *Tex19.1*^*−/−*^ spermatocytes have skewed recombination profiles. **a** Immunostaining for γH2AX (green) in *Tex19.1*^*+/±*^ and *Tex19.1*^*−/−*^ autosomally synapsed pachytene spermatocyte chromosome spreads. SYCP3 (red) marks the lateral element of the synaptonemal complex. A single channel greyscale image for γH2AX is also shown. The *Tex19.1*^*+/±*^ image belongs to the intermediate γH2AX foci category (1–40 S-foci), the *Tex19.1*^*−/−*^ image belongs to the high γH2AX foci category (> 40 S-foci). Asterisks indicate sex bodies, and arrowheads indicate example S-foci associated with axes. Scale bars 10 μm. **b** Quantification of γH2AX S-foci; 172 and 120 autosomally synapsed *Tex19.1*^*+/±*^ and *Tex19.1*^*−/−*^ pachytene nuclei were categorised according to the number of γH2AX S-foci; 22.1, 62.2 and 15.7% of pachytene *Tex19.1*^*+/±*^ spermatocytes have > 40, 1–40 and 0 γH2AX foci, respectively, compared to 52.3, 38.5 and 9.2% *Tex19.1*^*−/−*^ pachytene nuclei. Asterisks denote significance (*χ*^2^ test, ***p < 0.001). Nuclei were obtained from three animals for each genotype, and circles and vertical lines represent the means and interquartile distances of animals within each genotype. **c** Immunostaining for MLH1 (green) in control and *Tex19.1*^*−/−*^ autosomally synapsed pachytene spermatocyte chromosome spreads. SYCP3 (red) marks the lateral element of the synaptonemal complex. Examples of MLH1-positive and MLH1-negative images are shown. Nuclei containing ten or more axis-associated MLH1-foci (arrowheads) were classified as MLH1-positive. Scale bars 10 μm. **d** Quantification of pachytene nuclei that are MLH1-positive; 162 autosomally synapsed pachytene nuclei were scored for each genotype; 61.1% *Tex19.1*^*+/±*^ and 19.8% *Tex19.1*^*−/−*^ nuclei were MLH1-positive. Asterisks denote significance (*χ*^2^ test, ***p < 0.001). Nuclei were obtained from three animals for each genotype, and circles and vertical lines represent the means and interquartile distances of animals within each genotype

To verify our analysis of early recombination intermediates in pachytene *Tex19.1*^*−/−*^ spermatocytes, we examined the behaviour of γH2AX (Fig. 2a), a marker for unrepaired DNA damage (Mahadevaiah et al. 2001; Chicheportiche et al. 2007). In pachytene, γH2AX is typically detected as a strong cloud of staining over the asynapsed sex chromosomes, and as condensed axial foci (S-foci) on autosomes that co-localise with recombination foci (Chicheportiche et al. 2007). The number of autosomal γH2AX S-foci decreases during pachytene and become undetectable in diplotene (Chicheportiche et al. 2007). As distinguishing individual S-foci becomes somewhat subjective when there are large numbers of adjacent γH2AX S-foci on the same axis, we grouped nuclei as having abundant (> 40), intermediate (1–40) or no γH2AX S-foci. In control mice, 22% of autosomally synapsed pachytene spermatocytes had abundant, 62% had intermediate and 16% had no γH2AX S-foci (Fig. 2b). Autosomally synapsed pachytene spermatocytes with abundant γH2AX S-foci were ~ 2-fold more frequent in *Tex19.1*^*−/−*^ mice, and correspondingly fewer autosomally synapsed *Tex19.1*^*−/−*^ pachytene nuclei had intermediate or no γH2AX S-foci (Fig. 2b). Loss of *Tex19.1* does not have a detectable effect on the frequency of *Spo11*-independent γH2AX foci in spermatocytes (Crichton et al. 2017), suggesting that the increase in the number of pachytene spermatocytes with abundant γH2AX S-foci represents a difference in behaviour of *Spo11*-dependent meiotic DSBs in these mutants. Therefore, in addition to containing elevated levels of early recombination foci, autosomally synapsed *Tex19.1*^*−/−*^ pachytene spermatocytes more frequently contain abundant γH2AX S-foci suggesting there is more unrepaired DNA damage in these nuclei.

### Late recombination foci are depleted in autosomally synapsed *Tex19.1*^*−/−*^ pachytene spermatocytes

We next tested whether the increased frequency of early recombination intermediates and unrepaired DNA damage in pachytene *Tex19.1*^*−/−*^ spermatocytes is accompanied by changes in the frequency of spermatocytes containing markers of late recombination foci. Autosomally synapsed pachytene *Tex19.1*^*−/−*^ spermatocytes were scored for the presence or absence of MLH1 (Fig. 2c), a component of late recombination foci that marks crossover recombination events (Anderson et al. 1999). Consistent with the ability of *Tex19.1*^*−/−*^ spermatocytes to form functional chiasmata (Öllinger et al. 2008), MLH1 foci were readily detectable in autosomally synapsed *Tex19.1*^*−/−*^ spermatocytes (Fig. 2c). However, while 61% of control pachytene nuclei displayed MLH1 foci associated with autosomal axes, this was reduced to just 20% among pachytene *Tex19.1*^*−/−*^ nuclei (Fig. 2d). Thus, the population of autosomally synapsed pachytene *Tex19.1*^*−/−*^ spermatocytes has more early recombination foci, is enriched for nuclei containing large numbers of γH2AX S-foci and is depleted for nuclei containing late recombination foci.

### Histone H1t expression is delayed in autosomally synapsed *Tex19.1*^*−/−*^ spermatocytes

**Fig. 3:**
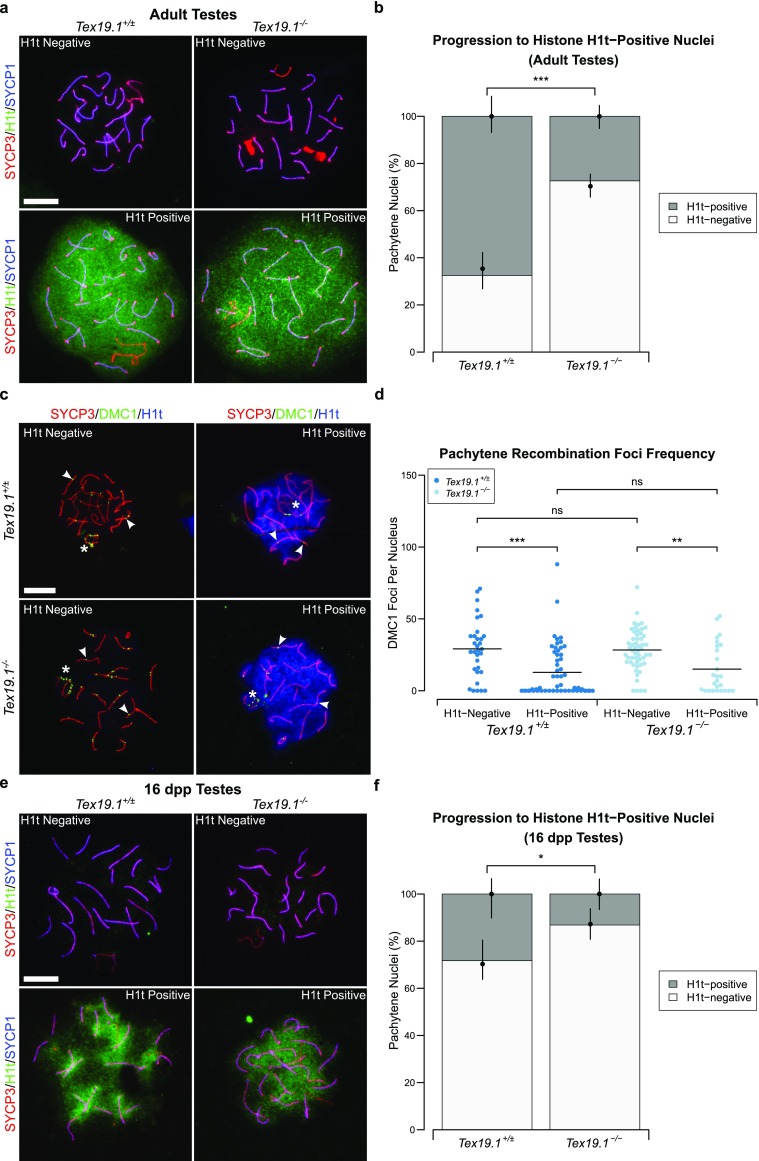
Expression of the mid/late pachytene marker histone H1t is delayed in autosomally synapsed pachytene *Tex19.1*^*−/−*^ spermatocytes. **a** Immunostaining for histone H1t (green) in *Tex19.1*^*+/±*^ and *Tex19.1*^*−/−*^ autosomally synapsed pachytene spermatocyte chromosome spreads. Examples of histone H1t-positive and H1t-negative images are shown. SYCP3 (red) and SYCP1 (blue) mark the lateral elements and transverse filaments of the synaptonemal complex, respectively. Scale bars 10 μm. **b** Quantification of the proportions of autosomally synapsed pachytene nuclei that are H1t-positive; 67.6% of *Tex19.1*^*+/±*^ and 27.4% of *Tex19.1*^*−/−*^ pachytene nuclei (*n* = 111, 95; Fisher’s exact test, *** indicates *p* < 0.001) obtained from three animals of each genotype were H1t-positive. **c** Co-immunostaining for DMC1 (green) and histone H1t (blue) in *Tex19.1*^*+/±*^ and *Tex19.1*^*−/−*^ autosomally synapsed pachytene spermatocyte chromosome spreads. SYCP3 (red) marks the lateral elements of the synaptonemal complex. DMC1 foci co-localising with axes were scored as recombination foci. Asterisks indicate sex chromosomes, arrowheads indicate example recombination foci. Scale bar 10 μm. **d** Scatterplot showing the number of axial recombination foci in H1t-positive and H1t-negative autosomally synapsed pachytene nuclei from *Tex19.1*^*+/±*^ and *Tex19.1*^*−/−*^ testes. Means are indicated by horizontal lines. Mean foci frequencies are 29 ± 20, 13 ± 18, 28 ± 14 and 15 ± 17 from left to right across the plot. Numbers of nuclei analysed were 32, 55, 53 and 26 from a total of at least 3 experimental or 3 control animals. Foci counts were compared using a Mann-Whitney *U* test; asterisks denote significance (**p < 0.01, ***p < 0.001), ns indicates no significant difference (*p* > 0.05). **e** Immunostaining for histone H1t (green) in autosomally synapsed pachytene spermatocyte chromosome spreads from 16 dpp prepubertal *Tex19.1*^*+/±*^ and *Tex19.1*^*−/−*^ animals. Examples of histone H1t-positive and H1t-negative images are shown; SYCP3 (red) and SYCP1 (blue) mark the lateral elements and transverse filaments of the synaptonemal complex, respectively. Scale bars 10 μm. **f** Quantification of the proportions of autosomally synapsed pachytene nuclei at 16 dpp that are H1t-positive; 28.3% of *Tex19.1*^*+/±*^ and 13.2% of *Tex19.1*^*−/−*^ pachytene nuclei (*n* = 99, 76; Fisher’s exact test, * indicates *p* < 0.05) obtained from three animals of each genotype were H1t-positive

Immunostaining for the mid/late pachytene marker histone H1t (Cobb et al. 1999; Moens et al. 2002) has previously been used to monitor spermatocyte progression through pachytene (Barchi et al. 2005; Mahadevaiah et al. 2008; Broering et al. 2014; Pacheco et al. 2015; Marcet-Ortega et al. 2017). Therefore, we next assessed whether the altered recombination kinetics in autosomally synapsed pachytene *Tex19.1*^*−/−*^ spermatocytes are associated with changes in histone H1t immunostaining. Histone H1t immunostaining is present throughout the chromatin of pachytene spermatocytes (Fig. 3a), and while 68% of control pachytene spermatocytes express histone H1t, only 27% of autosomally synapsed pachytene *Tex19.1*^*−/−*^ spermatocytes express this marker (Fig. 3b). Therefore, the abnormal recombination profile seen in *Tex19.1*^*−/−*^ spermatocytes is accompanied by changes in the proportion of pachytene cells expressing a marker of mid/late pachytene spermatocytes. This was previously reported to be a feature of the recombination-dependent pachytene arrest that occurs in *Trip13*^*mod/mod*^ spermatocytes (Pacheco et al. 2015).

We next investigated how DMC1 foci frequency relates to the presence of histone H1t in control and *Tex19.1*^*−/−*^ autosomally synapsed pachytene spermatocytes by co-immunostaining meiotic chromosome spreads with antibodies to histone H1t and antibodies to DMC1 (Fig. 3c). Histone H1t-positive control pachytene spermatocytes had approximately half the mean number of DMC1 foci than histone H1t-negative control pachytene spermatocytes (Fig. 3d). These data are consistent with cells progressing from high to low numbers of DMC1 foci and from a H1t-negative to a H1t-positive state as they advance through pachytene. However, the overlap in range of DMC1 foci between the histone H1t-positive and histone H1t-negative populations suggests that the absolute number of DMC1 foci likely does not determine the spermatocytes’ transition from H1t-negative to H1t-positive. Autosomally synapsed *Tex19.1*^*−/−*^ spermatocytes show a similar reduction in DMC1 foci frequency between histone H1t-negative and histone H1t-positive nuclei as seen in the control pachytene spermatocytes (Fig. 3d). Interestingly, although autosomally synapsed *Tex19.1*^*−/−*^ pachytene spermatocytes have more DMC1 foci that control pachytene spermatocytes (Fig. 1d), the number of DMC1 foci in H1t-negative autosomally synapsed *Tex19.1*^*−/−*^ pachytene spermatocytes is not significantly different from the number of DMC1 foci in H1t-negative autosomally synapsed control pachytene spermatocytes (Fig. 3d). Similarly, the number of DMC1 foci in H1t-positive autosomally synapsed *Tex19.1*^*−/−*^ pachytene spermatocytes is not significantly different from the number of DMC1 foci in H1t-positive autosomally synapsed control pachytene spermatocytes (Fig. 3d). These data suggest that the increased number of DMC1 foci in autosomally synapsed *Tex19.1*^*−/−*^ pachytene spermatocytes (Fig. 1) reflects an over-representation of spermatocytes with characteristics of early pachytene and that there might be some coupling between the progression of meiotic recombination and the expression or incorporation of histone H1t into meiotic chromatin during pachytene.

We next investigated further whether the over-representation of synapsed pachytene spermatocytes with immature characteristics in *Tex19.1*^*−/−*^ mice might be caused by cell death skewing the distribution of the pachytene population. Chromosome axes are insufficiently preserved in TUNEL-positive *Tex19.1*^*−/−*^ nuclei to assess whether there is elevated cell death within the synapsed pachytene population in these testes (Öllinger et al. 2008). However, increased cell death among *Tex19.1*^*−/−*^ spermatocytes does not take place until after 16 days post-partum (dpp) (Öllinger et al. 2008). We therefore determined whether the enrichment of immature pachytene spermatocytes is independent of cell death in *Tex19.1*^*−/−*^ testes by investigating histone H1t expression in 16 dpp pachytene *Tex19.1*^*−/−*^ spermatocytes. TUNEL staining on 16 dpp testis sections confirmed that there is no increase in cell death in *Tex19.1*^*−/−*^ spermatocytes relative to *Tex19.1*^*+/±*^ spermatocytes at this stage (Online Resource 1). Immunostaining of spermatocyte chromosome spreads from the same animals revealed that 28% of *Tex19.1*^*+/±*^ pachytene spermatocytes express histone H1t, while only 13% of autosomally synapsed pachytene *Tex19.1*^*−/−*^ spermatocytes express this marker (Fig. 3e, f). Therefore, the reduction in the proportion of pachytene *Tex19.1*^*−/−*^ spermatocytes expressing histone H1t does not appear to be caused by death of more mature pachytene cells, or an indirect consequence of cell death in the asynapsed pachytene cells in the same testicular environment.

**Fig. 4:**
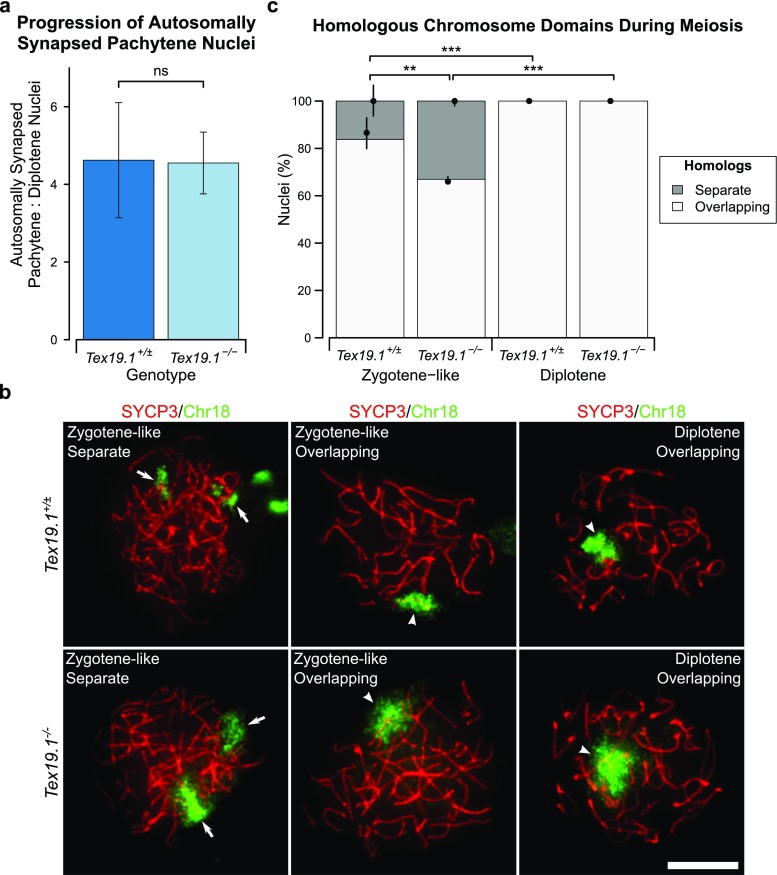
Autosomally synapsed, but not asynapsed, *Tex19.1*^*−/−*^ spermatocytes progress to diplotene. **a** Ratio of autosomally synapsed pachytene to diplotene nuclei in meiotic chromosome spreads from *Tex19.1*^*+/±*^ and *Tex19.1*^*−/−*^ testes. Ratios are derived from the meiotic progression data shown in Online Resource 1 and were obtained from a total of 462 *Tex19.1*^*+/±*^ and 459 *Tex19.1*^*−/−*^ nuclei from three animals for each genotype. ns indicates no significant difference (4.6 ± 1.5, 4.5 ± 0.8; *p* = 0.94, Student’s *t* test). **b** ImmunoFISH for chromosome 18 in zygotene-like and diplotene *Tex19.1*^*+/±*^ and *Tex19.1*^*−/−*^ spermatocytes. SYCP3 (red) marks the lateral element of the synaptonemal complex, the chromosome 18 paint is shown in green. The two homologues were scored as having overlapping (arrowheads) or separate (arrows) chromatin domains. Chromosome 18 FISH signals from an adjacent SYCP3-negative nucleus are visible in the top left panel. Scale bar 10 μm. **c** Quantification of chromosome domain configurations in *Tex19.1*^*+/±*^ and *Tex19.1*^*−/−*^ spermatocytes; 273 *Tex19.1*^*+/±*^ and 335 *Tex19.1*^*−/−*^ spermatocyte nuclei at zygotene-like or diplotene stage of meiosis I were categorised. FISH data for chromosome 18 and chromosome 19 were similar between genotypes and meiotic stage and were combined to provide more statistical power; 16.2% of 102 *Tex19.1*^*+/±*^ zygotene-like nuclei and 33.1% of 121 *Tex19.1*^*−/−*^ zygotene-like nuclei had separate chromosome domains, and all diplotene nuclei had overlapping domains. Asterisks denote significance (Fisher’s exact test, **p < 0.01, ***p < 0.001). Nuclei were obtained from three animals for each genotype, and circles and vertical lines represent the means and interquartile distances of animals within each genotype

Furthermore, if there is significant cell death in the autosomally synapsed pachytene *Tex19.1*^*−/−*^ population that contributes to the skew towards markers of early pachytene, this might be expected to decrease the proportion of fully synapsed pachytene cells that progress to diplotene. The ratio of autosomally synapsed pachytene to diplotene spermatocytes is not significantly altered in *Tex19.1*^*−/−*^ mice relative to *Tex19.1*^*+/±*^ controls (Fig. 4a, Online Resource 1), suggesting that the fully synapsed pachytene *Tex19.1*^*−/−*^ population is not beset with extensive cell death. However, while extensive autosomal asynapsis typically triggers cell death in pachytene in stage IV tubules (reviewed in Burgoyne et al. 2009), a single asynapsed copy of an additional non-essential chromosome is not sufficient to trigger spermatocyte death in pachytene in stage IV tubules (Mahadevaiah et al. 2008). Therefore, it is possible that some asynapsed pachytene *Tex19.1*^*−/−*^ spermatocytes are able to progress to diplotene and mask a decrease in the autosomally synapsed pachytene to diplotene ratio in this analysis. Approximately one third of pachytene *Tex19.1*^*−/−*^ spermatocytes are reported to have low level asynapsis involving up to five pairs of autosomes (Yang et al. 2010), and in the genetic background studied here, 28% of asynapsed pachytene *Tex19.1*^*−/−*^ spermatocytes have only one pair of asynapsed autosomes (Online Resource 1). To test whether *Tex19.1*^*−/−*^ spermatocytes with low level asynapsis might be able to progress into diplotene, we used immunoFISH to visualise chromosome domains in meiotic chromosome spreads. SYCP3 immunostaining in these experiments allowed us to identify diplotene nuclei and zygotene-like nuclei. These zygotene-like cells encompass zygotene and asynapsed pachytene cells that can be better distinguished in conventional SYCP3/SYCP1 co-immunostaining of meiotic chromosome spreads. ImmunoFISH for chromosome 18 or chromosome 19, the two smallest mouse autosomes, showed that separate non-overlapping chromosome FISH domains are present in 16% of zygotene-like spermatocytes from control *Tex19.1*^*+/±*^ testes, but never in diplotene control spermatocytes (Fig. 4b, c). This suggests that zygotene-like spermatocytes that have not paired their chromosome 18 or chromosome 19 homologues are either eliminated or pair these homologues before progression to diplotene. Interestingly, separate non-overlapping chromosome FISH domains were observed in 33% of zygotene-like *Tex19.1*^*−/−*^ spermatocytes, a higher proportion than in zygotene-like *Tex19.1*^*+/±*^ spermatocytes which presumably reflects the increased frequency of asynapsis in *Tex19.1*^*−/−*^ spermatocytes (Fig. 4b, c). However, separate non-overlapping chromosome FISH domains were not detected in diplotene *Tex19.1*^*−/−*^ spermatocytes (Fig. 4b, c). Therefore, although asynapsis in pachytene *Tex19.1*^*−/−*^ spermatocytes commonly involves only a small number of autosomes, we have not been able to detect any diplotene nuclei with non-overlapping homologous autosome domains.

Asynapsed autosomes can co-localise with sex chromosomes and become incorporated into the sex body (Mahadevaiah et al. 2008), which could potentially cause chromosome domains to overlap in the absence of synapsis. However, the single autosome domains that covered two separate SYCP3-containing chromosome axes in diplotene only occasionally overlapped the sex body and with similar frequencies in *Tex19.1*^*+/±*^ and *Tex19.1*^*−/−*^ spermatocytes (4/69 and 5/71 nuclei, respectively, no significant difference, Fisher’s exact test). Although we cannot exclude the possibility that some asynapsed pachytene spermatocytes with specific chromosome configurations can progress to diplotene in *Tex19.1*^*−/−*^ testes, this does not appear to be a frequent occurrence. The similarity in the ratio of autosomally synapsed pachytene to diplotene spermatocytes between *Tex19.1*^*+/±*^ and *Tex19.1*^*−/−*^ mice (Fig. 4a) therefore likely reflects similar viability and temporal progression of *Tex19.1*^*−/−*^ and control autosomally synapsed pachytene spermatocytes. Thus, we have been unable to detect any evidence of cell death in fully synapsed *Tex19.1*^*−/−*^ spermatocytes that could cause over-representation of early pachytene markers in this population. Rather the enrichment of early features of pachytene in fully synapsed *Tex19.1*^*−/−*^ spermatocytes might be caused by delayed progression of these spermatocytes through early pachytene.

### Abnormalities in histone H1t expression and γH2AX distribution are present in *Tex19.1*^*−/−*^ pachytene spermatocytes in late stage seminiferous tubules

**Fig. 5:**
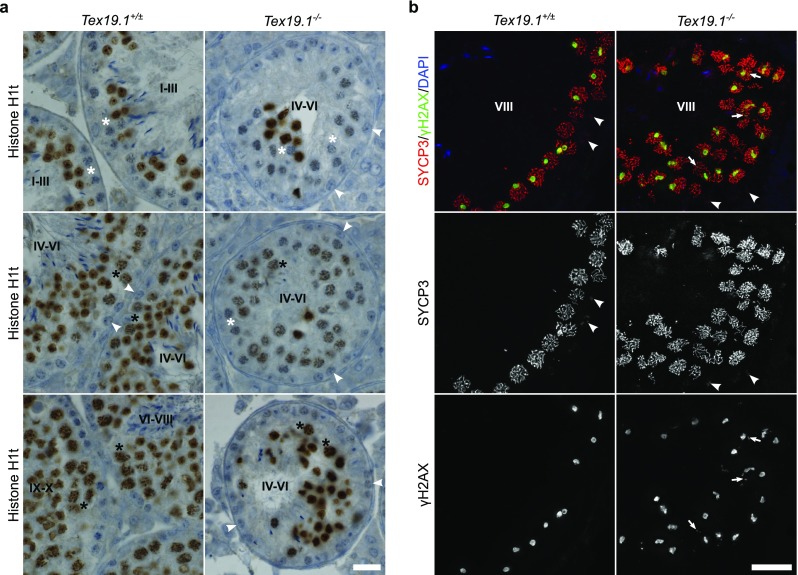
Features of early pachytene persist in late stage *Tex19.1*^*−/−*^ seminiferous tubules. **a** Immunohistochemistry for histone H1t (brown) in *Tex19.1*^*+/±*^ and *Tex19.1*^*−/−*^ testis sections counterstained with haematoxylin. Testis tubules were staged on the basis of the types of spermatocyte and spermatogonia present (Ahmed and de Rooij 2009). Tubule stages are indicated with Roman numerals and example histone H1t-negative pachytene spermatocytes (white asterisks), histone H1t-positive pachytene spermatocytes (black asterisks) and B spermatogonia (white arrowheads) are annotated. Tubules containing B-spermatogonia (late stage IV to early stage VI) only contained H1t-positive pachytene spermatocytes in *Tex19.1*^*+/±*^ testis sections, but two thirds of tubules containing B spermatogonia from *Tex19.1*^*−/−*^ testis had some H1t-negative pachytene spermatocytes. Ten tubules containing B spermatogonia were scored for each of three mice for each genotype. *Tex19.1*^*−/−*^ tubules have fewer post-meiotic spermatids and smaller diameters than control *Tex19.1*^*+/±*^ tubules (Öllinger et al. 2008). Scale bar 20 μm. **b** Immunofluorescence for the synaptonemal complex component SYCP3 (red) and the DNA damage marker γH2AX (green) in *Tex19.1*^*+/±*^ and *Tex19.1*^*−/−*^ testis sections counterstained with DAPI (blue) to visualise DNA. 2D projections of deconvolved z-stacks are shown. Greyscale images of SYCP3 and γH2AX are shown below the merged image. Preleptotene spermatocytes (arrowheads) in stage VIII tubules were identified by aggregates of SYCP3 and a lack of γH2AX in spermatocytes around the edge of the tubules (Mahadevaiah et al. 2001; Nakata et al. 2015). γH2AX staining was assessed in the strongly SYCP3-positive pachytene spermatocytes lumenal to the preleptotene spermatocytes. γH2AX was typically present in a large domain likely corresponding to the sex body in these cells and, in approximately 30% of stage VIII tubules from *Tex19.1*^*−/−*^ testes, was also present as additional nuclear foci (arrowheads) in the pachytene spermatocytes. *Tex19.1*^*−/−*^ tubules have fewer post-meiotic spermatids and smaller diameters than control *Tex19.1*^*+/±*^ tubules (Öllinger et al. 2008). Scale bar 20 μm

To investigate further whether the changes in histone H1t immunostaining and recombination that we have detected in the autosomally synapsed *Tex19.1*^*−/−*^ spermatocyte population might represent delayed progression through early pachytene, we analysed these features in testis sections. As the spatial and temporal progression of spermatogenesis in the testis results in associations between specific substages of spermatogenesis within the seminiferous epithelium (Ahmed and de Rooij 2009), spermatocytes that have been in pachytene for different amounts of time can be identified by their association with seminiferous epithelium stages. Zygotene spermatocytes enter pachytene in stage XII of the seminiferous epithelium and progress through pachytene from stage I through to stage X where they enter diplotene (Ahmed and de Rooij 2009). Pachytene spermatocytes start to immunostain for histone H1t during stage IV, and histone H1t immunostaining persists throughout the rest of meiosis and into post-meiotic spermiogenesis (Drabent et al. 1996; Mahadevaiah et al. 2008). Histone H1t immunostaining of control *Tex19.1*^*+/±*^ testes sections resulted in the expected pattern of histone H1t immunostaining: pachytene spermatocytes in early tubule stages (I–III) were H1t-negative, and pachytene spermatocytes at later tubule stages (late stage IV to early stage X) were H1t-positive (Fig. 5a). Whereas all tubules with B spermatogonia (late stage IV to early stage VI) contained only H1t-positive pachytene spermatocytes in *Tex19.1*^*+/±*^ control testes, approximately two thirds of tubules with B spermatogonia (late stage IV to early stage VI) from *Tex19.1*^*−/−*^ testes contained H1t-negative pachytene spermatocytes (Fig. 5a). H1t-positive pachytene spermatocytes and H1t-positive post-meiotic spermatids were often also present in tubules with B spermatogonia in *Tex19.1*^*−/−*^ testes (Fig. 5a). Some stage IV–VI tubules had a mixture of H1t-positive and H1t-negative pachytene spermatocytes (Fig. 5a), suggesting that the lack of H1t staining in H1t-negative *Tex19.1*^*−/−*^ pachytene spermatocytes is a consequence of cell autonomous defects within the spermatocytes themselves, rather than an effect of the tubule environment. The presence of these H1t-negative pachytene spermatocytes at later tubule stages in *Tex19.1*^*−/−*^ testes suggests that H1t expression is perturbed in some *Tex19.1*^*−/−*^ spermatocytes during their progression through pachytene. It is not clear whether these H1t-negative *Tex19.1*^*−/−*^ pachytene spermatocytes have altered regulation of histone H1t at the level of transcription, RNA stability, translation or protein stability.

We next tested whether the increased autosome-associated γH2AX staining that we detected in chromosome spreads from *Tex19.1*^*−/−*^ testes (Fig. 2a, b) might also be detectable in pachytene spermatocytes in later tubule stages in testis sections. We used immunostaining for the synaptonemal complex component SYCP3 and the DNA damage marker γH2AX to identify preleptotene spermatocytes in stage VIII tubules (Mahadevaiah et al. 2001; Nakata et al. 2015), and assessed γH2AX staining in the pachytene spermatocytes in these tubules. In control *Tex19.1*^*+/±*^ testes, pachytene spermatocytes in stage VIII tubules typically contained a large prominent cloud of γH2AX staining representing the sex body and no detectable γH2AX staining elsewhere in the nucleus (Fig. 5b). However, around one third of the stage VIII tubules in *Tex19.1*^*−/−*^ testes contained pachytene spermatocytes with small nuclear foci of γH2AX in addition to the presumptive sex body (Fig. 5b). Therefore, some pachytene *Tex19.1*^*−/−*^ spermatocytes present at this late tubule stage have abnormal distributions of the early DNA damage marker γH2AX.

While we cannot exclude the possibility that some asynapsed pachytene spermatocytes in *Tex19.1*^*−/−*^ testes evade the synapsis checkpoint acting in stage IV tubules and contribute to the lack of histone H1t expression at stages IV–VI and abnormal γH2AX staining at stage VIII, both these phenotypes are consistent with changes in histone H1t expression and autosome-associated γH2AX foci seen in chromosome spreads of autosomally synapsed *Tex19.1*^*−/−*^ spermatocytes (Figs. 2a and 3a). Taken together, these alternative approaches suggest that autosomally synapsed *Tex19.1*^*−/−*^ spermatocytes in late pachytene are presenting features that are normally associated with earlier stages of pachytene.

### The *Tex19.1*^*−/−*^ pachytene spermatocyte phenotype is distinct from that of *Trip13*^*mod/mod*^

**Fig. 6:**
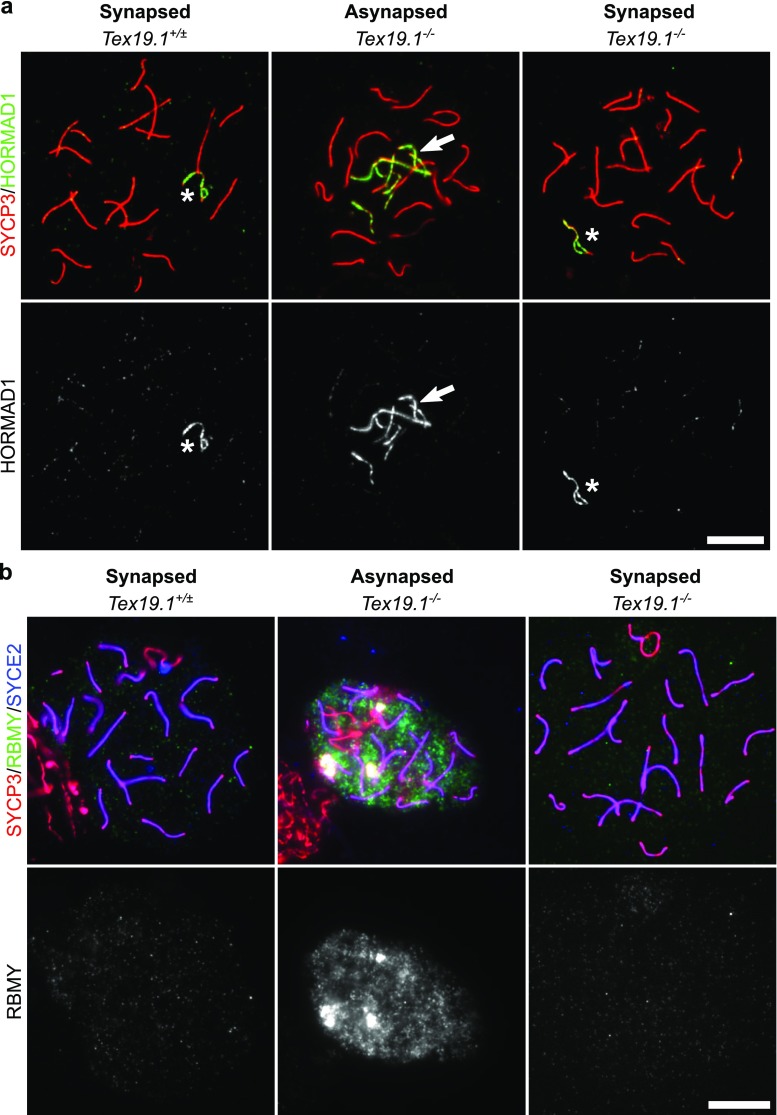
Autosomally synapsed pachytene *Tex19.1*^*−/−*^ spermatocytes correctly localise HORMAD1 and undergo functional MSCI. **a**, **b** Immunostaining for HORMAD1 (**a**, green) and RBMY (**b**, green) in *Tex19.1*^*+/±*^ and *Tex19.1*^*−/−*^ autosomally synapsed pachytene spermatocyte chromosome spreads. *Tex19.1*^*−/−*^ autosomally asynapsed pachytene spermatocytes showing localisation of HORMAD1 to asynapsed autosomes and failure to silence the Y-encoded RBMY protein are shown as positive controls. SYCP3 (**a**, **b**, red) and SYCE2 (**a**, blue) mark the lateral and central elements of the synaptonemal complex, respectively. The sex body is marked with an asterisk. Single channel greyscale images for HORMAD1 and RBMY are also shown. Regions of adjacent non-pachytene nuclei are visible in some of the panels in **b**. RBMY images are representative of 64 *Tex19.1*^*+/±*^ nuclei and 78 *Tex19.1*^*−/−*^ pachytene nuclei obtained from 3 animals of each genotype. HORMAD1 images are representative of 162 *Tex19.1*^*+/±*^ nuclei and 957 *Tex19.1*^*−/−*^ pachytene nuclei obtained from 3 *Tex19.1*^*+/±*^ and 3 *Tex19.1*^*−/−*^ animals. Scale bars 10 μm

Although *Trip13*^*mod/mod*^ and *Tex19.1*^*−/−*^ autosomally synapsed pachytene spermatocytes differ significantly in their ability to progress through pachytene into diplotene, these cells share some phenotypic features including elevated frequencies of unrepaired DSBs and histone H1t-negative cells (Pacheco et al. 2015) (Figs. 1, 2, 3 and 4). We therefore tested whether loss of *Tex19.1* might phenocopy other aspects of the *Trip13*^*mod/mod*^ spermatocyte phenotype. *Trip13*^*mod/mod*^ spermatocytes have defects in the removal of HORMAD proteins from synapsed chromosome axes (Wojtasz et al. 2009); therefore, we immunostained *Tex19.1*^*−/−*^ meiotic chromosome spreads for HORMAD1. As expected, HORMAD1 is absent from synapsed autosomes in wild-type spermatocytes but persists on the unsynapsed non-homologous arms of the sex chromosomes of synapsed spermatocytes. HORMAD1 was also detected along asynapsed regions of chromosome axes in asynapsed *Tex19.1*^*−/−*^ spermatocytes (Fig. 6a); therefore, in contrast to *Trip13*^*mod/mod*^ spermatocytes, HORMAD1 appears to be removed normally from synapsed axes in *Tex19.1*^*−/−*^ spermatocytes (Fig. 6a).

*Trip13*^*mod/mod*^ pachytene spermatocytes have also been reported to exhibit defects in MSCI (Pacheco et al. 2015). Introducing mutations in the *Chk2*-dependent DNA damage signalling pathway allows *Trip13*^*mod/mod*^ spermatocytes to progress to a histone H1t-positive state, but these spermatocytes arrest in pachytene with defects in MSCI (Pacheco et al. 2015; Marcet-Ortega et al. 2017). To test whether MSCI is being functionally established in *Tex19*.*1*^*−/−*^ spermatocytes, we immunostained meiotic chromosome spreads for RBMY, a Y-chromosome-encoded protein silenced by MSCI during pachytene (Turner et al. 2002). RBMY was readily detected in autosomally asynapsed pachytene *Tex19.1*^*−/−*^ spermatocytes but not in either control or *Tex19.1*^*−/−*^ autosomally synapsed pachytene spermatocytes (Fig. 6b). This suggests that MSCI is functioning in autosomally synapsed spermatocytes in the absence of *Tex19.1*.

Therefore, the phenotypic overlap of *Tex19.1*^*−/−*^ spermatocytes and *Trip13*^*mod/mod*^ spermatocytes does not extend to defective HORMAD1 dissociation or MSCI failure and pachytene cell death but is largely restricted to an elevation in unrepaired DSBs in pachytene and reduced histone H1t expression. These shared features between *Tex19.1*^*−/−*^ and *Trip13*^*mod/mod*^ spermatocytes could potentially represent recombination-dependent control of pachytene progression.

### Chromosome-associated post-translational modifications are altered in *Tex19.1*^*−/−*^ spermatocytes

**Fig. 7:**
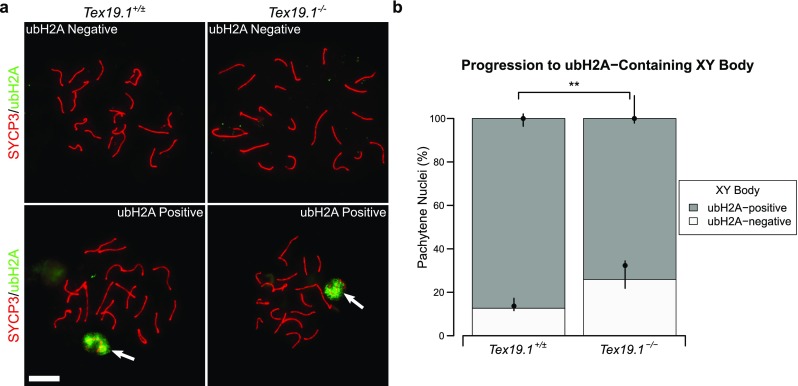
Autosomally synapsed pachytene *Tex19.1*^*−/−*^ spermatocytes are enriched for histone ubiquitylation patterns associated with early pachytene. **a** Immunostaining for ubiquitylated H2A (ubH2A, green) in *Tex19.1*^*+/±*^ and *Tex19.1*^*−/−*^ autosomally synapsed pachytene spermatocyte chromosome spreads. Examples of ubH2A-positive and ubH2A-negative images are shown. ubH2A clouds associating with the XY body are indicated by arrows. SYCP3 (red) marks the lateral element of the synaptonemal complex. Scale bars 10 μm. **b** Quantification of the proportions of autosomally synapsed pachytene nuclei that have ubH2A clouds associated with the XY body; 87.3% of *Tex19.1*^*+/±*^ and 74.1% of *Tex19.1*^*−/−*^ pachytene nuclei (*n* = 158, 147; Fisher’s exact test, ** indicates *p* < 0.01) obtained from four animals of each genotype have XY-associated ubH2A

A number of post-translational histone modifications occur as mouse spermatocytes progress through meiotic prophase (Crichton et al. 2014). Therefore, we used these to assess pachytene progression in autosomally synapsed pachytene *Tex19.1*^*−/−*^ spermatocytes further. Ubiquitylated histone H2A (ubH2A) accumulates at the sex body, and although it is not required for MSCI, the change in ubH2A distribution during pachytene is a potential marker to monitor pachytene progression (Baarends et al. 2005; Lu et al. 2010). Immunostaining control spermatocytes for ubH2A revealed a cloud of staining at the sex chromosomes (Fig. 7a), similar to that previously reported (Baarends et al. 2005; Lu et al. 2010), in 87% of control pachytene spermatocytes (Fig. 6b). A cloud of ubH2A staining was also detected at the sex chromosomes in autosomally synapsed *Tex19.1*^*−/−*^ pachytene spermatocytes (Fig. 7a); however, the proportion of cells with such staining was reduced to 74% (Fig. 7b). Thus, the delay in maturation of recombination in autosomally synapsed *Tex19.1*^*−/−*^ pachytene spermatocytes is accompanied by more widespread changes in progression through pachytene.

**Fig. 8:**
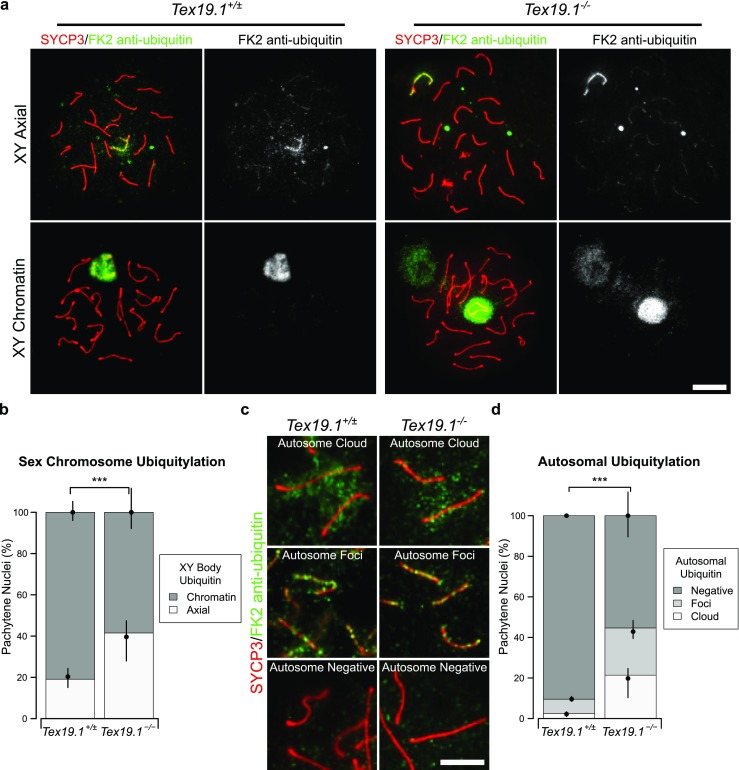
Early pachytene chromatin-associated polyubiquitylation patterns are enriched in autosomally synapsed *Tex19.1*^*−/−*^ spermatocytes **a** Immunostaining for mono- and poly-ubiquitylated proteins (FK2 anti-ubiquitin, green) in *Tex19.1*^*+/±*^ and *Tex19.1*^*−/−*^ autosomally synapsed pachytene spermatocyte chromosome spreads. Examples of nuclei with axial and chromatin staining on the XY body are shown. SYCP3 (red) marks the lateral element of the synaptonemal complex. A single channel greyscale image for FK2 is also shown. An adjacent non-meiotic nucleus positive for FK2 is visible in the XY chromatin *Tex19.1*^*−/−*^ panel. Scale bar 10 μm. **b** Quantification of the proportions of autosomally synapsed pachytene nuclei with different sex chromosome ubiquitylation patterns; 19.1% of *Tex19.1*^*+/±*^ and 41.5% of *Tex19.1*^*−/−*^ pachytene nuclei obtained from three animals of each genotype have axial staining on the XY body (*n* = 157, 159; *χ*^2^ test, *** indicates *p* < 0.001). **c** Higher magnification and increased exposure of FK2 immunostaining showing examples of staining on autosomal axes. Examples of autosomes with clouds, foci and no FK2 staining are shown. SYCP3 (red) marks the lateral element of the synaptonemal complex. Scale bar 5 μm. **d** Quantification of the proportions of autosomally synapsed pachytene nuclei with clouds, foci or no FK2 staining on their autosomes. Autosomally synapsed pachytene nuclei were scored for autosomal FK2 staining regardless of the FK2 staining pattern on the sex chromosomes; 2.6% of *Tex19.1*^*+/±*^ and 21.4% of *Tex19.1*^*−/−*^ pachytene nuclei obtained from three animals of each genotype have clouds of FK2 staining on their autosomal axes, and 7.0% of *Tex19.1*^*+/±*^ and 23.3% of *Tex19.1*^*−/−*^ pachytene nuclei have foci of FK2 staining on their autosomal axes (*n* = 157, 159; *χ*^2^ test, *** indicates *p* < 0.001)

In addition to the specific establishment of H2A ubiquitylation at the sex body, the patterns of total ubiquitylation associated with chromosomes also vary as spermatocytes progress through pachytene (An et al. 2012; Rao et al. 2017). Ubiquitylation can be broadly monitored using the FK2 monoclonal antibody which recognises both mono- and poly-ubiquitylation (Fujimuro et al. 1994). Therefore, we investigated the progression of ubiquitylation in *Tex19.1*^*−/−*^ by immunostaining spermatocyte chromosome spreads with the FK2 antibody. Consistent with previous reports, FK2 staining in control pachytene spermatocytes is strikingly enriched at the sex chromosomes (Fig. 8a) (An et al. 2012; Rao et al. 2017). FK2 staining is initially restricted to the chromosome axes in the XY body in early pachytene, then extends throughout the XY body chromatin in mid and late pachytene (An et al. 2012; Rao et al. 2017). Although both control and *Tex19.1*^*−/−*^ autosomally synapsed pachytene spermatocytes exhibit enriched FK2 staining on the sex chromosomes (Fig. 8a), the frequency of pachytene nuclei with axial XY staining characteristic of early pachytene is higher in autosomally synapsed *Tex19.1*^*−/−*^ spermatocytes (Fig. 8b). Thus, similar to the recombination markers, γH2AX foci, histone H1t expression, and ubH2A patterns, autosomally synapsed *Tex19.1*^*−/−*^ spermatocytes are enriched for nuclei that display sex chromosome ubiquitylation patterns consistent with earlier substages of pachytene.

We also analysed the FK2 staining patterns on autosomes in autosomally synapsed pachytene *Tex19.1*^*−/−*^ spermatocytes. FK2 staining is weaker on the autosomes than on the sex chromosomes of pachytene spermatocytes (Fig. 8a), and autosomally synapsed pachytene spermatocytes were analysed for autosomal FK2 staining regardless of the FK2 staining pattern present on their sex chromosomes. FK2 typically stains axial foci during early-mid pachytene, then becomes more diffusely localised throughout autosomal chromatin as pachytene progresses (Rao et al. 2017). This accumulation of ubiquitylation on autosomal foci during early-mid pachytene potentially reflects the role of the ubiquitin proteasome system in maturation of recombination foci (Rao et al. 2017). The frequency of autosomally synapsed pachytene nuclei with axial FK2 staining on autosomes in foci or in a dense cloud (Fig. 8c) is greatly increased in *Tex19.1*^*−/−*^ mice (Fig. 8d). Therefore, the altered patterns of ubiquitylation in *Tex19.1*^*−/−*^ spermatocytes are not limited to ubH2A at the sex body but extend to more general ubiquitylation at the sex chromosomes and autosomes.

### *Tex19.1*^*−/−*^ spermatocytes have reduced chromosome axis elongation during pachytene

**Fig. 9:**
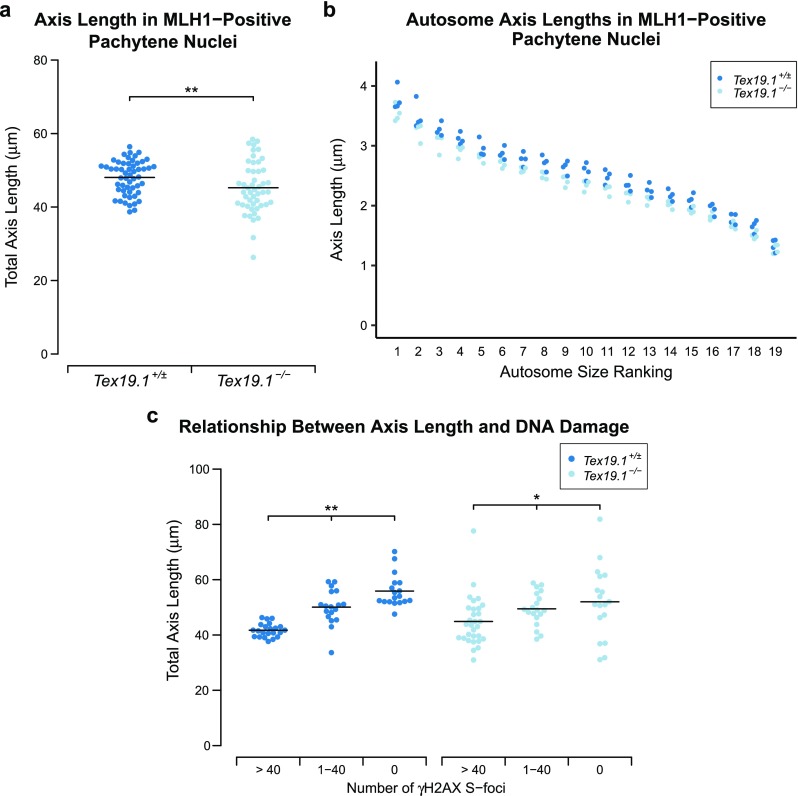
Axis elongation during pachytene is perturbed in autosomally synapsed pachytene *Tex19.1*^*−/−*^ spermatocytes. **a** Quantification of chromosome axis length in MLH1-positive autosomally synapsed *Tex19.1*^*+/±*^ and *Tex19.1*^*−/−*^ pachytene spermatocytes. Axis length was measured from SYCP3 immunostaining of chromosome spreads. The total length of all 19 autosomes for individual nuclei is shown, and the mean values for each genotype (48.0 ± 4.6 and 45.2 ± 7.0 μm) are indicated with a horizontal line. A total of 54 and 49 nuclei from four *Tex19.1*^*+/±*^ and *Tex19.1*^*−/−*^ animals, respectively, were scored; ** indicates *p* < 0.01 (Mann-Whitney *U* test). **b** Axis lengths (μm) of individual size-ranked autosomes from MLH1-positive autosomally synapsed *Tex19.1*^*+/±*^ and *Tex19.1*^*−/−*^ pachytene spermatocytes shown in **a**. **c** Plot showing the relationship between γH2AX S-foci and axis elongation in autosomally synapsed *Tex19.1*^*+/±*^ and *Tex19.1*^*−/−*^ pachytene spermatocytes. Total axis lengths for individual nuclei belonging to the indicated categories of γH2AX S-foci are plotted. In contrast to **a**, these nuclei were not selected for being MLH1-positive. Means for each group are indicated by horizontal lines (41.7 ± 2.5, 50.1 ± 6.3, 55.9 ± 5.9, 44.9 ± 8.9, 49.5 ± 6.0 and 52.0 ± 12.9 μm from left to right, *n* = 22, 18, 18, 30, 19 and 18, respectively). Statistically significant differences between groups were determined by Mann-Whitney *U* test; ** indicates *p* < 0.01, * indicates *p* < 0.05. None of the comparisons between the *Tex19.1*^*+/±*^ and *Tex19.1*^*−/−*^ groups are significantly different. Nuclei were derived from three control animals and four *Tex19.1*^*−/−*^ animals

The data on recombination and DNA damage markers, histone H1t and ubiquitylation together indicate that fully synapsed pachytene *Tex19.1*^*−/−*^ nuclei might be delayed in their progression from early to mid/late pachytene. However, it is not clear whether the autosomally synapsed *Tex19.1*^*−/−*^ spermatocytes that reached late pachytene were still delayed in their meiotic progression. Progression through pachytene is reported to be associated with elongation of the autosomal chromosome axes between early and late pachytene (Vranis et al. 2010); therefore, we used chromosome axis length to assess whether late pachytene *Tex19.1*^*−/−*^ nuclei are still delayed in aspects of their meiotic progression. The late recombination marker MLH1 was used to select for spermatocytes in late pachytene for this analysis. Comparison of total axis length between *Tex19.1*^*+/±*^ and *Tex19.1*^*−/−*^ MLH1-positive autosomally synapsed spermatocytes revealed a 6.5% reduction in axis length in the absence of *Tex19.1* (Fig. 9a, Table 1). Comparisons between axes ranked by size, as has been performed in similar analyses (Roig et al. 2010), demonstrated that all chromosome sizes were similarly affected (Fig. 9b, Table 1). A significant reduction in length was not achieved for the smallest group of axes, though this is likely due to a greater degree of measurement error relative to total length for shorter axes. Thus, loss of *Tex19.1* results in chromosomes having shorter axes in late pachytene spermatocytes.

**Table 1:** Axis lengths of groups of size-ranked autosomes in MLH1-positive autosomally synapsed pachytene nuclei

Grouped axis ranks	Mean group axis length (arbitrary units)		Difference in *Tex19.1*^−/−^ (%)
	*Tex19.1* ^+/±^	*Tex19.1* ^−/−^	
1–2	7.30	6.79	− 7.0*
3–5	9.39	8.80	− 6.3*
6–11	15.94	14.89	− 6.6*
12–16	10.72	9.98	− 6.9*
17–19	4.72	4.48	− 5.1
Total	48.07	44.94	− 6.5**

Autosomes were ranked by size and grouped as indicated. The mean total axis lengths for each group of autosomes are shown. Four animals were analysed for each genotype and a total of 50 and 55 nuclei analysed for *Tex19.1*^*+/±*^ and *Tex19.1*^*−/−*^, respectively. Student’s *t* test was used to test for statistical significance between genotypes (* indicates *p* < 0.05, ** indicates *p* < 0.01)

To determine whether loss of *Tex19.1* results in shorter chromosome axes independently of altered progression through pachytene, we classified the pachytene nuclei based on the frequency of γH2AX S-foci and compared axis length within and between groups. *Tex19.1*^*+/±*^ and *Tex19.1*^*−/−*^ spermatocytes both elongated their chromosome axes as they progressed through pachytene and repaired γH2AX S-foci (Fig. 9c). However, when autosomally synapsed *Tex19.1*^*+/±*^ and *Tex19.1*^*−/−*^ pachytene spermatocytes with similar amounts of DNA damage are compared, no significant differences in axis length are evident (Fig. 9c). Therefore, the shorter chromosome axes in MLH1-positive *Tex19.1*^*−/−*^ pachytene spermatocytes likely reflect the perturbed progression of these spermatocytes through pachytene.

### Delayed progression through pachytene in *Tex19.1*^*−/−*^ spermatocytes is dependent on *Spo11*

**Fig. 10:**
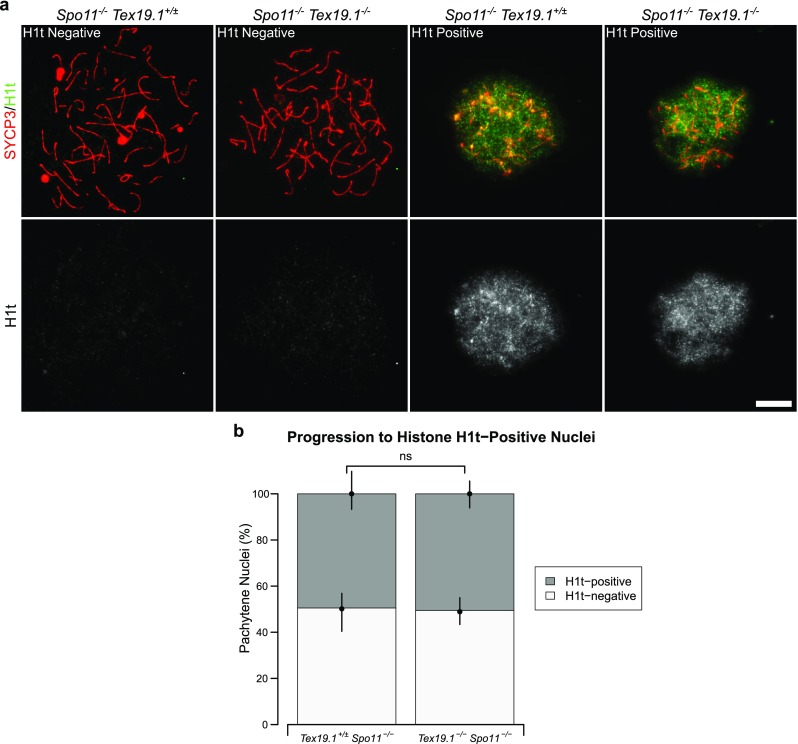
Delayed histone H1t expression in *Tex19.1*^*−/−*^ spermatocytes depends on *Spo11*. **a** Immunostaining for histone H1t (green) in *Spo11*^*−/−*^ *Tex19.1*^*+/±*^ single knockout and *Spo11*^*−/−*^ *Tex19.1*^*−/−*^ double knockout zygotene-like spermatocyte chromosome spreads. Example histone H1t-positive and H1t-negative images are shown. SYCP3 (red) marks the lateral element of the synaptonemal complex respectively. Scale bars 10 μm. **b** Quantification of the proportions of zygotene-like nuclei that are H1t-positive; 49.5% of *Spo11*^*−/−*^ *Tex19.1*^*+/±*^ and 50.5% of *Spo11*^*−/−*^ *Tex19.1*^*−/−*^ zygotene-like nuclei obtained from three animals of each genotype were H1t-positive (*n* = 97, 99; *χ*^2^ test, ns indicates no significant difference, *p* > 0.05)

Taken together, the data presented in this manuscript suggests that multiple aspects of progression to late pachytene are delayed in autosomally synapsed *Tex19.1*^*−/−*^ mutants. This phenotype could potentially reflect the delayed accumulation of early recombination foci in zygotene *Tex19.1*^*−/−*^ spermatocytes and the activity of a checkpoint co-ordinating progression through pachytene with repair of DNA damage or maturation of recombination foci. Alternatively, the altered progression through pachytene in autosomally synapsed *Tex19.1*^*−/−*^ spermatocytes could potentially reflect a recombination-independent role for *Tex19.1* in pachytene progression. To distinguish these possibilities, we assessed progression through pachytene in *Tex19.1*^*−/−*^ spermatocytes in the absence of meiotic DSBs. *Spo11*^*−/−*^ spermatocytes fail to generate meiotic DSBs and as such are unable to undergo meiotic recombination and arrest in a zygotene-like state with defective chromosome synapsis (Baudat et al. 2000; Romanienko and Camerini-Otero 2000). Despite this, *Spo11*^*−/−*^ spermatocytes are able to progress to a mid/late pachytene-like state of gene expression and recruit histone H1t (Barchi et al. 2005). Therefore, we tested whether the delay in histone H1t expression seen in pachytene *Tex19.1*^*−/−*^ spermatocytes depends on the presence of *Spo11* and meiotic recombination. Consistent with a previous report (Barchi et al. 2005), we detected H1t staining in approximately 50% of zygotene-like control *Spo11*^*−/−*^ mutant spermatocytes (Fig. 10a, b). In *Tex19.1*^*−/−*^ *Spo11*^*−/−*^ double knockout mice, a similar frequency of zygotene-like spermatocytes was H1t-positive (Fig. 10a, b). Therefore, the reduced progression of *Tex19.1*^*−/−*^ spermatocytes to an H1t-positive mid/late pachytene-like state of gene expression is dependent on the meiotic DSB forming endonuclease *Spo11*. This finding indicates that the delayed progression through pachytene observed in *Tex19.1*^*−/−*^ spermatocytes is related to the meiotic recombination defects incurred in this mutant.

## Discussion

**Fig. 11:**
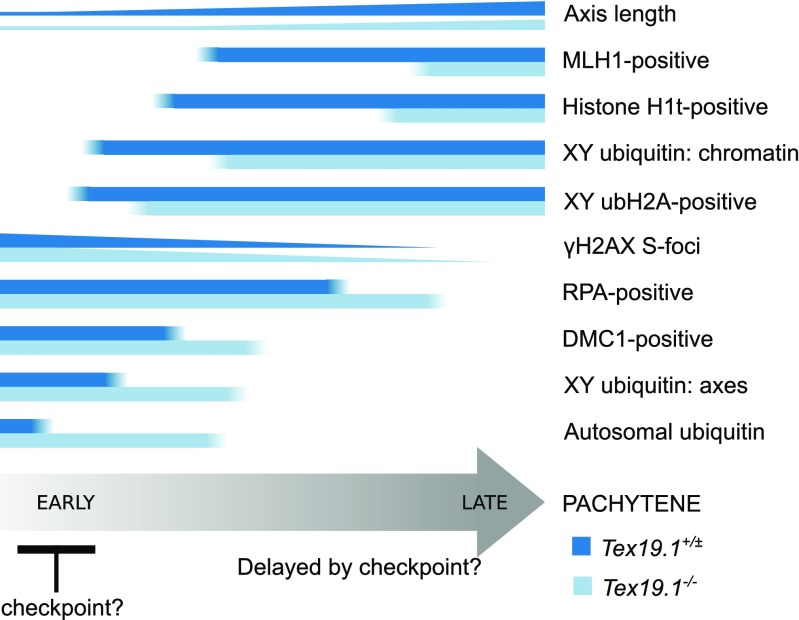
Schematic diagram showing delayed progression through pachytene in autosomally synapsed pachytene *Tex19.1*^*−/−*^ spermatocytes. Summary of aspects of progression through pachytene analysed in this study. Horizontal bars indicate the proportion of pachytene nuclei expressing different markers of progression through pachytene. Number of γH2AX foci and length of axis are indicated by the height of the bars. Proportions of DMC1 and RPA-positive nuclei are estimated from the violin plots in Online Resource 1. The differences in the behaviour of these markers in autosomally synapsed pachytene *Tex19.1*^*+/±*^ and *Tex19.1*^*−/−*^ spermatocytes are indicated by the blue bars. *Tex19.1*^*−/−*^ spermatocytes appear to activate a *Spo11*-dependent recombination checkpoint in early pachytene that delays progression to late pachytene

A recombination-dependent block in synapsed pachytene spermatocyte development has previously been reported in *Trip13*^*mod/mod*^ hypomorphic mutant mice with defects in the repair of meiotic DSBs (Pacheco et al. 2015). In this study, we have investigated whether delayed meiotic recombination has consequences for progression through pachytene in mouse spermatocytes using an independent mouse mutant containing some pachytene spermatocytes that complete chromosome synapsis but have defects in recombination. The data presented in this study show that autosomally synapsed *Tex19.1*^*−/−*^ spermatocytes have perturbed progression through pachytene (Fig. 11). It is possible that some or even all aspects of this perturbed pachytene progression reflect a direct role for *Tex19.1* in regulating these processes. However, our finding that the ability of *Tex19.1* to alter histone H1t expression depends on *Spo11* is more consistent with *Tex19.1* influencing pachytene progression indirectly through a recombination-dependent checkpoint that co-ordinately regulates aspects of progression through pachytene. Other aspects of the skewed progression of *Tex19.1*^*−/−*^ spermatocytes are possibly more likely to reflect substrate-product relationships between the delayed accumulation of early recombination foci in zygotene and their subsequent maturation rather than regulation by a recombination-dependent checkpoint. For example, the enrichment of early recombination markers, seen in autosomally synapsed *Tex19.1*^*−/−*^ spermatocytes during pachytene, could be a direct consequence of the delayed accumulation of early recombination foci in zygotene. Indeed, mouse spermatocytes with altered *Spo11* expression have a delayed burst of DSB formation in zygotene that also translates into an enrichment for early recombination markers during pachytene (Faieta et al. 2016). Further work is needed to elucidate the pathway(s) that link the *Tex19.1*^*−/−*^ mutation to the multiple aspects of pachytene progression that are affected in these mutants.

Our finding that delayed histone H1t expression in pachytene in *Tex19.1*^*−/−*^ spermatocytes depends on *Spo11* is consistent with meiotic recombination defects in *Tex19.1*^*−/−*^ spermatocytes activating a potential recombination-dependent checkpoint in pachytene. However, we do not currently know the nature of the *Spo11*-dependent lesion that might be activating a recombination-dependent checkpoint in pachytene *Tex19.1*^*−/−*^ spermatocytes. Histone H1t expression is commonly used to assess how far mutant spermatocytes progress through pachytene (Barchi et al. 2005; Mahadevaiah et al. 2008; Pacheco et al. 2015). However, our analysis of *Tex19.1*^*−/−*^ spermatocytes suggests that the timing of histone H1t expression within pachytene may not be fixed and could be influenced by the recombination intermediates or lesions. It is possible that some of the differences in histone H1t expression between mutant spermatocytes which exhibit cell death in stage IV tubules such as *Spo11*^*−/−*^, *Dmc1*^*−/−*^, *Msh5*^*−/−*^ and *Trip13*^*mod/mod*^ (Barchi et al. 2005; Mahadevaiah et al. 2008; Pacheco et al. 2015) could at least partly reflect the presence or absence of recombination intermediates or lesions that trigger this checkpoint to regulate histone H1t expression. Our analysis suggests that although there is some coupling between progression of meiotic recombination and histone H1t expression that could potentially reflect a normal recombination intermediate indirectly regulating H1t expression through a checkpoint-like mechanism, H1t expression is not strictly dependent on a deterministic threshold DMC1 foci frequency. Further studies will be required to determine whether other markers of meiotic recombination intermediates correlate better with histone H1t expression. Normal recombination intermediates could have deleterious effects if they persist during the meiotic nuclear divisions (Copsey et al. 2013), and it is possible that a recombination-dependent checkpoint may have a role in co-ordinating progression through pachytene during normal meiosis in wild-type cells by delaying aspects of progression through pachytene until recombination proceeds beyond a certain point. Indeed, *H2afx* and *Mdc1*, which are involved in DNA damage responses in somatic cells, have recently been reported to influence the timing of histone H1t expression in mouse spermatocytes without defective recombination (Testa et al. 2018). Alternatively, *Tex19.1*^*−/−*^ spermatocytes could have additional defects in processing recombination intermediates, and abnormal lesions arising during maturation of recombination intermediates could be activating a recombination-dependent checkpoint.

Despite completing autosomal synapsis, moderate severity *Trip13* mutants exhibit defects in dissociation of HORMAD1 from synapsed autosome axes, defects in processing recombination intermediates and extensive cell death in histone H1t-negative pachytene spermatocytes (Li et al. 2007; Wojtasz et al. 2009; Roig et al. 2010; Pacheco et al. 2015). There are clear distinctions between the *Trip13* phenotype and the phenotype of autosomally synapsed *Tex19.1*^*−/−*^ pachytene spermatocytes: autosomally synapsed *Tex19.1*^*−/−*^ spermatocytes do not phenocopy autosomally synapsed *Trip13*^*mod/mod*^ spermatocytes with respect to HORMAD1 dissociation and do not undergo extensive cell death during pachytene. The difference in dissociation of HORMAD1 from synapsed chromosome axes between these mutants could potentially have consequences for DSB repair (Wojtasz et al. 2009; Rinaldi et al. 2017; Carofiglio et al. 2018). Furthermore, although both *Trip13*^*mod/mod*^ and *Tex19.1*^*−/−*^ spermatocytes exhibit increased numbers of RAD51/DMC1 and RPA foci on synapsed autosomes, it is not clear whether this represents similar defects in recombination in these mutants. Leptotene *Tex19.1*^*−/−*^ spermatocytes have reduced amounts of γH2AX and reduced numbers of RAD51, DMC1 or RPA-containing early recombination foci that then increase in frequency in zygotene suggesting that many of the recombination foci present in pachytene may be delayed (Crichton et al. 2017). *Trip13*^*mod/mod*^ spermatocytes also have delayed accumulation of RAD51/DMC1 foci during leptotene and zygotene, but there is no detectable change in γH2AX abundance at these stages and increased numbers of RPA foci during leptotene suggesting that loading of early recombination proteins onto resected DSBs is perturbed (Roig et al. 2010). *Trip13*^*mod/mod*^ spermatocytes also have defects in progression of recombination during pachytene (Li et al. 2007; Roig et al. 2010). It is not clear whether the recombination-dependent delay in histone H1t expression that we have described in autosomally synapsed *Tex19.1*^*−/−*^ spermatocytes is activated in *Trip13* mutant spermatocytes, or whether the ATM-dependent DNA damage signalling cascade activated in autosomally synapsed *Trip13*^*mod/mod*^ spermatocytes (Pacheco et al. 2015; Marcet-Ortega et al. 2017) is also activated in autosomally synapsed *Tex19.1*^*−/−*^ spermatocytes. Analysis of transgenic mouse models that have altered *Spo11* expression and delayed DSB formation (Faieta et al. 2016) may help to determine whether delays in early meiotic recombination are sufficient to trigger a recombination-dependent delay in histone H1t expression during pachytene, and investigating the role of the ATM-dependent DNA damage signalling cascade in progression of autosomally synapsed *Tex19.1*^*−/−*^ spermatocytes through pachytene might help to determine whether a similar DNA damage response is being triggered in both *Tex19.1*^*−/−*^ and *Trip13*^*mod/mod*^ spermatocytes.

Autosomally synapsed *Trip13*^*mod/mod*^ hypomorphic spermatocytes are reported to undergo cell death in pachytene before histone H1t is expressed, while inactivation of *Atm* in this genetic background results in cell death and MSCI defects in histone H1t-positive pachytene spermatocytes (Pacheco et al. 2015; Marcet-Ortega et al. 2017). Our findings in *Tex19.1*^*−/−*^ synapsed pachytene spermatocytes provide support from a different mouse model that defects in meiotic recombination can impact on pachytene progression independently of any detectable effect on synapsis. They also extend the role of any potential recombination-dependent checkpoint beyond acting as a quality control to eliminate spermatocytes with defective recombination to modulating meiotic progression to allow for DSB repair and spermatocyte survival (Fig. 11). Although there are examples of checkpoints inducing pachytene delays in yeast (Xu et al. 1997), worms (Carlton et al. 2006) and flies (Joyce and McKim 2010), defects in synapsis or recombination have only been reported to trigger cell death in pachytene mouse spermatocytes (Burgoyne et al. 2009; Pacheco et al. 2015). The change in progression through pachytene in response to delayed recombination that we have identified would potentially allow, or reflect the repair, of recombination-dependent lesions in these cells before they continue further through meiosis.

## Electronic supplementary material

ESM 1 (PDF 930 kb)
