# Supplementary material for: Defects in meiotic recombination delay progression through pachytene in Tex19.1−/− mouse spermatocytes
Source: Chromosoma. 2018 Jun 16;127(4):437–59. doi: 10.1007/s00412-018-0674-9 (PMC6208735; doi:10.1007/s00412-018-0674-9)
Supplement: Supplementary file 1 — (PDF 930 kb) [file 412_2018_674_MOESM1_ESM.pdf]

Online Resource 1

Chromosoma

Defects in Meiotic Recombination Delay Progression Through Pachytene in *Tex19.1*$^{-/-}$ Mouse Spermatocytes

James H. Crichton, David Read [†], and Ian R. Adams [*]

MRC Human Genetics Unit, MRC Institute of Genetics and Molecular Medicine, University of Edinburgh, Western General Hospital, Crewe Road, Edinburgh, UK. EH4 2XU.

[†] Deceased

[*] Author for correspondence:

Email: Ian.Adams@igmm.ed.ac.uk

Tel: +44 131 332 2471

ORCID: 0000-0001-8838-1271

Contents

Supplementary Files and Tables

Supplementary Table S1

Primary Antibodies Used for Immunostaining Meiotic Chromosome Spreads

Supplementary Fig. S1

Distribution of Recombination Foci Numbers in Pachytene *Tex19.1*$^{-/-}$ Spermatocytes

Supplementary Fig. S2

Cell Death in *Tex19.1*$^{-/-}$ 16 dpp Testes

Supplementary Fig. S3

Progression of *Tex19.1*$^{-/-}$ Spermatocytes Through Meiosis

Supplementary Fig. S4

Asynapsis in *Tex19.1*$^{-/-}$ Spermatocytes

Antibody	Species	Source	Dilution
Anti-DMC1	Rabbit	Santa Cruz, H-100, sc-22768	1:50
Anti-RAD51	Rabbit	Calbiochem, PC130	1:500
Anti-RPA	Rabbit	C. James Ingles, University of Toronto, Toronto, Canada	1:300
Anti-SYCE2	Guinea Pig	Howard Cooke, MRC HGU, Edinburgh, UK	1:1000
Anti-SYCP3	Mouse	Santa Cruz, D-1, sc-74569	1:200
Anti-SYCP3	Mouse	Abcam, ab97672	1:500
Anti-SYCP3	Rabbit	Abcam, ab1592	1:300
Anti-SYCP3	Rabbit	LS Bio, LS-B175	1:500
Anti-SYCP1	Rabbit	Abcam, ab15090	1:200
Anti-γH2AX	Mouse	Millipore, 05-636	1:2000
Anti-γH2AX	Rabbit	Millipore, 07-164	1:200
Anti-ubH2A	Mouse	Millipore, 05-678	1:200
FK2 anti-Ub	Mouse	Enzo Life Sciences, BML-PW8810	1:500
Anti-MLH1	Mouse	Pharmingen, 51-1327GR	1:50
Anti-RBMY	Rabbit	David Elliott, University of Newcastle, UK	1:100
Anti-HORMAD1	Guinea Pig	Attila Tóth, Technische Universität Dresden, Germany	1:200
Anti-H1t	Guinea Pig	Mary Ann Handel, Jackson Laboratory, Bar Harbor, USA	1:1000

Supplementary Table S1

Primary Antibodies Used for Immunostaining Meiotic Chromosome Spreads

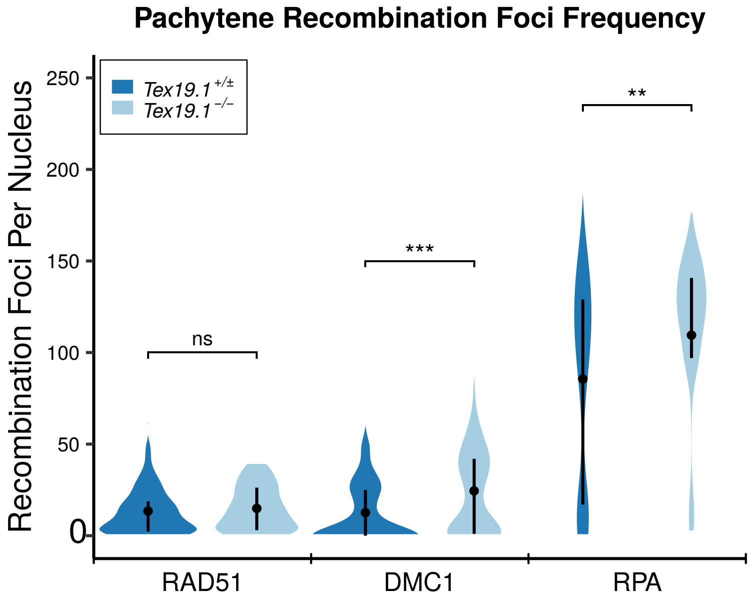

Supplementary Fig. S1

Distribution of Recombination Foci Numbers in Pachytene *Tex19.1*$^{-/-}$ Spermatocytes.

Violin plots showing the distribution of axial recombination foci in autosomally synapsed pachytene nuclei. Data are the same as those plotted in Figure 1D but re-plotted to allow the distributions to be visualised better. Violin plots are normalised so that the area of each plot is the same, and the width of each plot at a given height represents the proportion of nuclei containing that number of foci. Means are indicated by circles and interquartile distances by vertical lines. The number of nuclei analysed were 66, 60, 165, 163, 109, 94 from a total of at least 3 experimental or 3 control animals for each recombination protein. Foci counts were compared between genotypes using a Mann-Whitney U test, asterisks denote significance (** $p < 0.01$, *** $p < 0.001$), ns indicates no significant difference ($p > 0.05$).

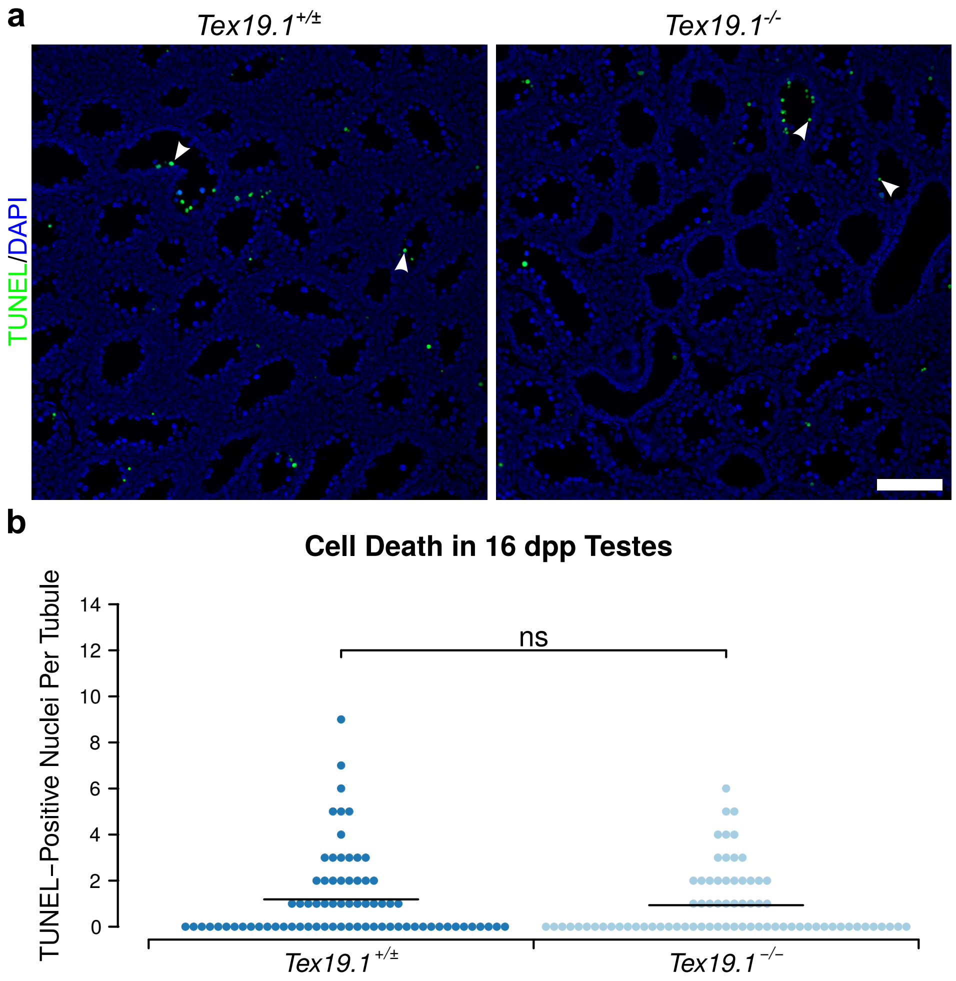

Supplementary Fig. S2

Cell Death in *Tex19.1*$^{-/-}$ 16 dpp Testes

a Identification of cells in testis sections undergoing cell death using TUNEL staining (green). Tissue was co-stained using DAPI (blue) to mark DNA. TUNEL-positive nuclei within seminiferous tubules (examples are indicated with arrowheads) were counted. Scale bar 100 µm. **b** Scatterplots showing the number of TUNEL-positive nuclei per seminiferous tubule cross section. TUNEL-positive cells were counted in 25 tubules from each of three *Tex19.1*$^{-/-}$ animals and three *Tex19.1*$^{+/\pm}$ littermate controls. Means for each genotype are indicated by horizontal lines (1.2±0.2, 0.9±0.2, n=75, 75). There is no statistically significant difference between genotypes (Mann-Whitney U test, p=0.45). ns indicates not significant.

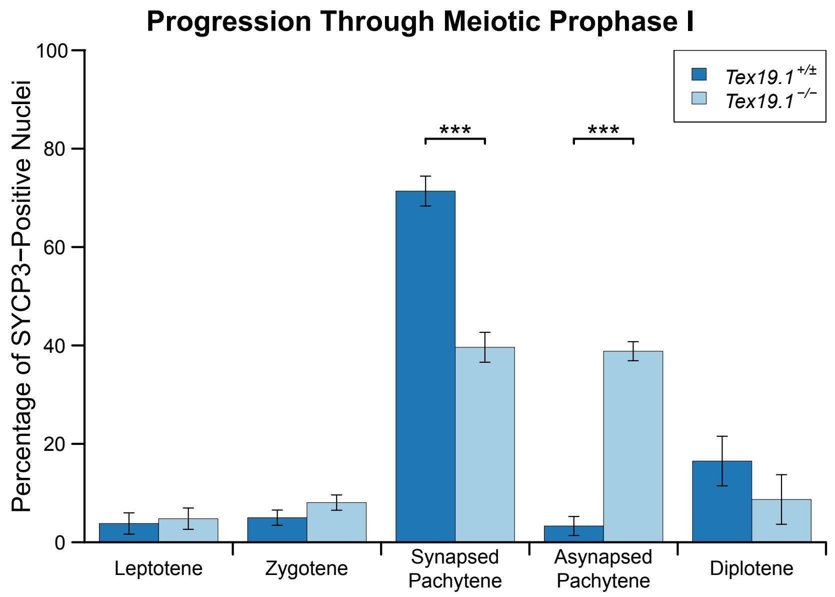

Supplementary Fig. S3

Progression of *Tex19.1*$^{-/-}$ Spermatocytes Through Meiosis.

Graph showing progression of *Tex19.1*$^{+/\pm}$ and *Tex19.1*$^{-/-}$ spermatocytes through meiotic prophase I. Meiotic chromosome spreads from *Tex19.1*$^{+/\pm}$ and *Tex19.1*$^{-/-}$ testes were immunostained for the synaptonemal complex components SYCP3 and SYCP1. SYCP3-positive nuclei were classified as leptotene, zygotene, pachytene, asynapsed pachytene or diplotene from low magnification images of random fields. Counts are based on a total of 462 *Tex19.1*$^{+/\pm}$ and 459 *Tex19.1*$^{-/-}$ nuclei from three animals for each genotype. *** indicates $p<0.001$ (Student's t-test).

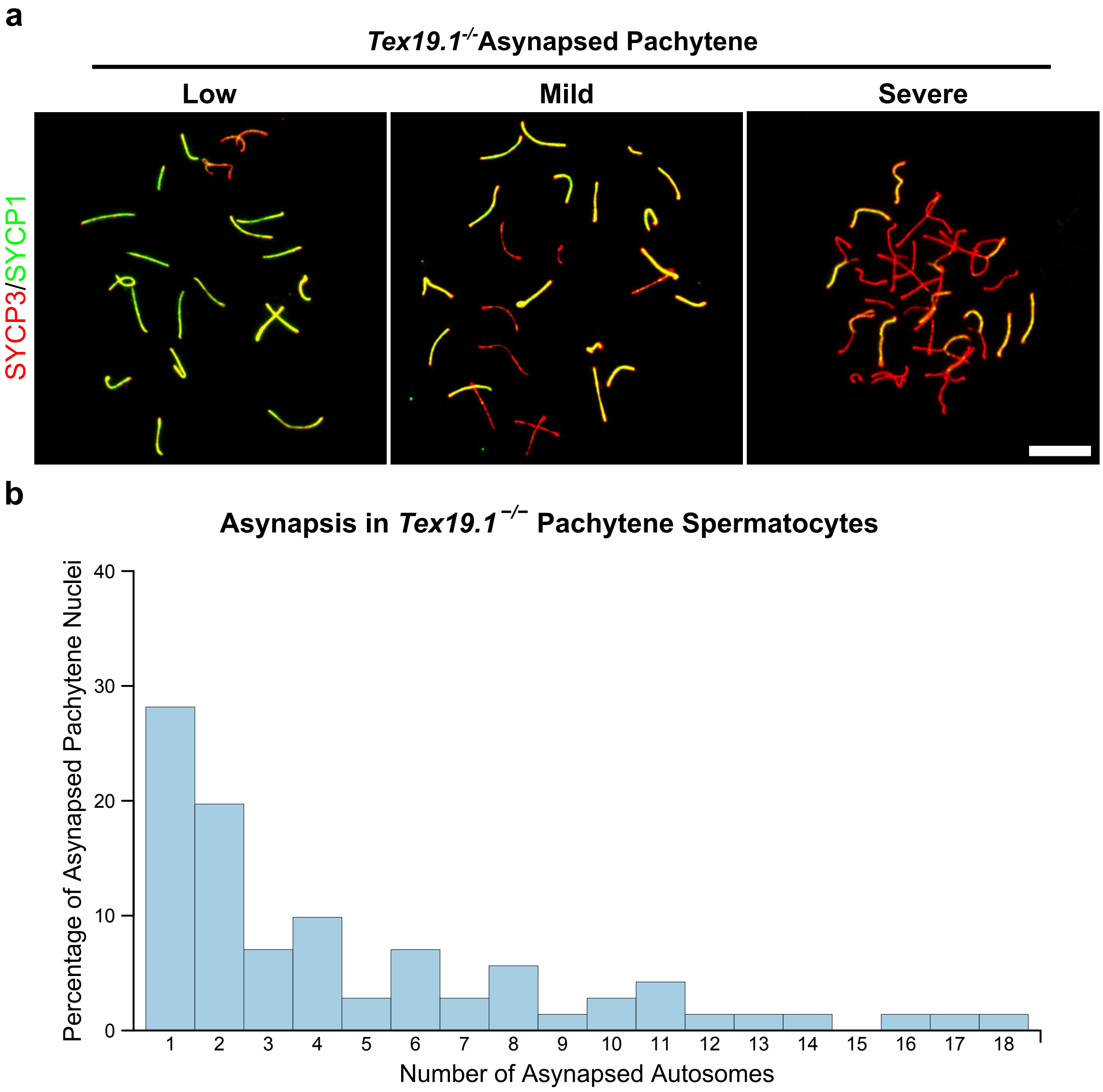

Supplementary Fig. S4

Asynapsis in *Tex19.1$^{-/-}$* Spermatocytes.

a Chromosome spreads from *Tex19.1$^{-/-}$* immunostained with synaptonemal complex markers SYCP3 and SYCP1 to assess synapsis. Synapsed axes contain SYCP1 and SYCP3, asynapsed axes only contain SYCP3. Examples of low, mild and severe asynapsis are shown. These images have 1, 3 and 14 asynapsed autosomes respectively. Asynapsed sex chromosomes may also be visible in these images. Scale bar 10 µm. **b** Histogram showing how frequently different severities of autosome asynapsis occurs in *Tex19.1$^{-/-}$* spermatocytes. Counts are derived from 71 asynapsed pachytene nuclei identified from a total of 147 pachytene nuclei.
